# Fossil record of stem groups employed in evaluating the chronogram of insects (Arthropoda: Hexapoda)

**DOI:** 10.1038/srep38939

**Published:** 2016-12-13

**Authors:** Yan-hui Wang, Michael S. Engel, José A. Rafael, Hao-yang Wu, Dávid Rédei, Qiang Xie, Gang Wang, Xiao-guang Liu, Wen-jun Bu

**Affiliations:** 1 College of Computer and Control Engineering, Nankai University, 38 Tongyan Road, Haihe Education Park, Jinnan District, Tianjin 300350, China; 2 Institute of Entomology, College of Life Sciences, Nankai University, 94 Weijin Road, Nankai District, Tianjin 300071, China; 3 Division of Entomology, Natural History Museum, and Department of Ecology & Evolutionary Biology, 1501 Crestline Drive – Suite 140, University of Kansas, Lawrence, Kansas 66045, USA; 4 Instituto Nacional de Pesquisas da Amazônia, INPA, Caixa Postal 478, 69011-970 Manaus, Amazonas, Brazil

## Abstract

Insecta *s. str.* (=Ectognatha), comprise the largest and most diversified group of living organisms, accounting for roughly half of the biodiversity on Earth. Understanding insect relationships and the specific time intervals for their episodes of radiation and extinction are critical to any comprehensive perspective on evolutionary events. Although some deeper nodes have been resolved congruently, the complete evolution of insects has remained obscure due to the lack of direct fossil evidence. Besides, various evolutionary phases of insects and the corresponding driving forces of diversification remain to be recognized. In this study, a comprehensive sample of all insect orders was used to reconstruct their phylogenetic relationships and estimate deep divergences. The phylogenetic relationships of insect orders were congruently recovered by Bayesian inference and maximum likelihood analyses. A complete timescale of divergences based on an uncorrelated log-normal relaxed clock model was established among all lineages of winged insects. The inferred timescale for various nodes are congruent with major historical events including the increase of atmospheric oxygen in the Late Silurian and earliest Devonian, the radiation of vascular plants in the Devonian, and with the available fossil record of the stem groups to various insect lineages in the Devonian and Carboniferous.

Over half of all described living species are insects, and they dominate all terrestrial ecosystems^1^. Insects are ancient, with definitive evidence of their occurrence as far back as the earliest Devonian, and they were the first lineage to evolve powered flight, a key innovation leading to subsequent phases of radiation and ecological specialization^1^,^2^,^3^,^4^. Understanding the factors that led to these episodes of diversification and extinction over the more than 400 million years of insect history is vital for any comprehensive perspective on their evolution. Crucial to this is a robust estimate of the timing of major events so that they may be placed within the proper paleoecological and paleoclimatological context. Unfortunately, the earliest fossils of insects have been shrouded in mystery, and only a few insights are available into the pre-Carboniferous hexapods^2^,^3^. Although insects have a rich fossil record, the vast majority of the materials come from the latest Carboniferous, Permian, and younger deposits^1^,^4^ and these postdate most of the more dramatic evolutionary events in insect history, such as the great radiation of winged lineages (the monophyletic Pterygota) that comprise more than 99% of all hexapod species^1^.

The earliest fossil attributed to an insect is a pair of mandibles preserved in the Pragian-aged Rhynie Chert beds of Scotland^2^. This species, *Rhyniognatha hirsti*, is a dicondylic metapterygotan insect, and thus places the origin of flight much earlier than previously thought and suggests that the first insects likely appeared during the Silurian^2^. Nonetheless, there remains a gap of approximately 100 million years between the earliest hexapods and their putative crustacean sister group, clade Xenocarida (=Remipedia + Cephalocarida)^5^,^6^ which has fossils at least as old as the Upper Cambrian, approximately 500 million years ago (Ma)^7^. Another lack of insect fossils between the Late Devonian and the early Serpukhovian (Hexapoda gap)^3^ renders determining the precise timing of origins and divergences of the main insect clades difficult.

In the absence of sufficient numbers of and suitable-preserved fossils, the molecular clock concept, viz., an estimation of divergence times based on molecular sequence data of extant organisms and calibrated against pertinent fossils belonging to the same higher-level taxa, allows a reconstruction of the timescale for the origin and early diversification of insects. Different positions of fossil species of a hypothetical clade are shown in Fig. 1. Only those that can be assigned with confidence to a certain extant taxon can be used in calibration (extinct groups 4–7). There are abundant fossils that do not belong to any extant orders, belong to a certain broadly defined clade (extinct group 1, such as the extinct order Palaeodictyoptera), or represent isolated lineages originating before divergences leading to extant groups (extinct groups 2 and 3). Although a “tip-dating” method has been proposed^8^,^9^ as a means of incorporating fossil ages into analyses, the potentially useful information provided by fossils has not been regularly utilized. Compilation of a morphological data matrix simultaneously for extant and fossil taxa of insects is exceedingly challenging owing to the rather fragmentarily preserved fossils, many times known only from isolated wings. Because of this limitation, only a handful of empirical studies have used this method^8^,^9^,^10^,^11^,^12^, and it is unlikely that it will be applicable for stem groups of insects. Furthermore, despite the considerable progress during the past few years, such as the establishment of relaxed and random clocks which made it possible to model molecular evolution with different rates along each lineage as well as between different lineages, some analytical biases can affect estimates of times of divergences. These include improper fossil calibration^13^,^14^, sparse taxonomic sampling^15^,^16^,^17^, or excessive restrictions on priors that can bias posterior estimates^18^,^19^.

Most previous studies estimating divergence times of insect clades focused on cladogenesis within particular orders, such as Coleoptera^20^, Diptera^21^, Lepidoptera^22^, Neuropterida (=Raphidioptera + Megaloptera + Neuroptera)^23^, Strepsiptera^24^, Hymenoptera^9^, and Hemiptera-Heteroptera^25^. Two significant works based on transcriptome datasets explored the times of origin for the main clades of Arthropoda^17^,^26^; these also included divergence dates for winged insects and some insect orders, but with an unsatisfying taxon sampling across the supercohort Polyneoptera. A recent phylogenomic study based on transcriptome data presented divergence time estimations for insects^27^, but congruence with other types of evidence needs to be further assessed.

Due to the improved taxon sampling and/or a better alignment of the molecular sequences, the results of different phylogenetic studies of insects during the past decade have reached a rough congruence in respect to several details^27^,^28^,^29^,^30^,^31^,^32^, such as the monophyly of Palaeoptera^27^,^30^,^33^,^34^, the ordinal relationships within Holometabola^27^,^35^,^36^,^37^, and the recognition of monophyletic groups within Polyneoptera^30^,^32^. Although a recent study investigated the phylogeny of insects based on transcriptome data^27^, it still suffered from some unusual groupings, most notably the sister-group relationship between Psocodea and Holometabola, rendering Paraneoptera paraphyletic.

In this study we utilized complete length sequences of five nuclear genes and thirteen mitochondrial protein-coding genes (PCGs) of representatives of all extant orders to reconstruct an ordinal-level phylogeny of insects. A complete timescale of insect divergences was then reconstructed based on a phylogenetic context, with high congruence with other various kinds of evidence in the case of the deeper nodes. Our results of divergence times correlate nicely with the fossil record of stem groups and certain paleoecological events.

## Results

### Phylogenetic relationships based on nuclear genes

The results of Bayesian inference (BI) and maximum likelihood (ML) analyses based on five nuclear genes are summarized in Fig. 2 and Supplementary Fig. S1. The results obtained from different algorithms largely came to the same topology. The monophyly of Dicondylia, Neoptera, Polyneoptera, and Holometabola received almost full support in BI and ML analyses. Within the heterogeneous Polyneoptera the previously proposed clades of Dermoplecopterida, Dictyoptera, Notoptera (=Mantophasmatodea + Grylloblattodea), and Eukinolabia (=Phasmatodea + Embiodea) received both high posterior probabilities and high bootstrap values. Blattopterida (=Cursorida, comprising Zoraptera + Dictyoptera) received support of 100% posterior probability (PP) and moderate bootstrap values. The clade Mecynoptera (=(Phasmatodea + Embiodea) + (Grylloblattodea + Mantophasmatodea)) was supported with high posterior probabilities in the BI analysis. The sister relationship between Thysanoptera and Hemiptera and the monophyly of Paraneoptera (=Acercaria, not including Zoraptera) were supported with moderate posterior probabilities in the BI analysis.

Considering the impact of the secondary structure on phylogenetic reconstruction, the topologies obtained in BI and ML analyses were also the same. Additionally, in the results of BI analyses using Doublet model for the substitution of the paired sites of rRNAs, the monophyly of Eumetabola (=Paraneoptera + Holometabola) was supported with 100% PP. That is, ignoring secondary structures does not have a great impact on tree topology and branch lengths in the process of phylogenetic reconstruction. In the phylogenetic results obtained from maximum parsimony (MP) analyses many clades were also supported by high or moderate bootstrap values. As a summary, our phylogenetic results show a high congruence with several previous works involving various kinds of evidence^27^,^29^,^30^,^31^,^32^,^34^,^35^,^36^,^38^,^39^. This arguably provides a strong basis for an estimation of divergence times of the deeper nodes of insects.

### Saturation test for the mitochondrial PCGs

Saturation test of the third codon positions of concatenated mitochondrial PCGs indicated that the index of substitution saturation (Iss) was significantly higher than the critical value of the index of saturation (Iss. cAsym) (P < 0.01; NumOUT = 16 or 32). This result suggested that these positions experienced substitution saturation and thus they were useless for phylogenetic reconstruction^40^. For the first and second codon positions, the Iss were all lower than the critical values, therefore only the first two codon positions were used in the phylogenetic reconstruction.

### Phylogenetic relationships based on nuclear genes and mitochondrial PCGs

Phylogenetic results inferred from the combined datasets of nuclear genes and different codon positions or amino acids of mitochondrial PCGs (matrices 2–5) are presented in Supplementary Figs S2–S5. The results showed that the topologies based on different combined datasets can vary dramatically in respect of the deeper nodes depending on the different matrices or algorithms. For example, the monophyly of Condylognatha was not recovered consistently in the BI and ML analyses (Supplementary Figs S3–S5), and even the monophyly of Holometabola received no support according to the result based on matrix 5 (Supplementary Fig. S5). The long branches, i.e., Strepsiptera: Mengenillidae, Hemiptera: Aphididae, and Zoraptera: Zorotypidae, were always attracted together as a group in the results of all MP analyses. Considering the fact that convincing evidences are available in support of the monophyly of Mecynoptera and Condylognatha^27^,^30^,^32^,^38^, it can be concluded that no type of mitochondrial genes is appropriate to indicate ordinal level relationships of insects. Accordingly, only nuclear genes were used to estimate the divergence times in the present study.

### Estimated divergence times in the deeper nodes of Insecta

For the normal prior distributions in the uncorrelated log-normal relaxed clock model (UCL), the divergence times of the deeper nodes of Insecta show that the differences are relatively small for most nodes under three different settings for the standard deviation (SD) (Table 1). Besides, under the same uniform prior distributions, the discrepancies of median ages ranged from 0 to 5 Ma between the two modelling schemes for the stem-encoding regions of rDNAs, and the median ages for 7 out of 17 nodes were the same (Table 1), which also suggested that the RNA secondary structures may sometimes lead to biased time estimation but the impact is alleviated to a large extent^41^. Thereafter, to make the description easier to follow, we employed the results inferred from the UCL clock model and the SD values restricted to ±one Stage/Age with normal distribution. The maximum clade credibility chronogram is summarized in Fig. 3. The information of the span of the 95% highest posterior density (HPD) and the divergence time with the median value were given for each node.

The inferred results suggest that the origin of Pterygota is dated at about 413 Ma (95% HPD 425–399 Ma). Shortly after their origin, Pterygota began to radiate in the Early Devonian (401 Ma, 95% HPD 418–384 Ma), with the paleopterous and neopterous insects diverging in the Middle and Upper Devonian. The radiation of Polyneoptera began in the late Middle Devonian (355 Ma, 95% HPD 386–316 Ma), and the subsequent episode of diversification appears to have taken place in the mid-Pennsylvanian. The split between clades Notoptera and Eukinolabia occurred at 258 Ma (95% HPD 329–191 Ma), while according to these analyses Zoraptera diverged from Dictyoptera at 273 Ma (95% HPD 328–215 Ma).

Paraneoptera and Holometabola diverged in the late Devonian and likely both diversified during the Mississippian period. Within Paraneoptera, the split between Psocodea and Condylognatha occurred around the Devonian and Carboniferous boundary (357 Ma, 95% HPD 378–333 Ma). Soon after the origin of Condylognatha, stem-Thysanoptera and stem-Hemiptera diverged from each other during the Middle Mississippian (339 Ma, 95% HPD 359–317 Ma). The hyperdiverse holometabolan insects probably radiated around the earliest Mississippian. The Hymenopterida (=stem- and crown-Hymenoptera) diverged from their common ancestor with the remainder of Holometabola about 354 Ma (95% HPD 378–329 Ma), while the common ancestors of Amphiesmenoptera and Antliophora apparently diverged during the Pennsylvanian period (313 Ma, 95% HPD 344–292 Ma). The split between the clade Coleopterida (=Coleoptera + Strepsiptera) and Neuropterida occurred approximately around the same time (312 Ma, 95% HPD 334–294 Ma).

## Discussion

Apart from transcriptome data, molecular markers with complete sequence data of orthologous genes, i.e., two nuclear rDNAs, three nuclear PCGs, and thirteen mitochondrial genes, were included in this study for nearly all insect orders. Our study comprehensively reexamined the applicability of these markers in studies on insect phylogeny at the ordinal level.

Comparing to a recent phylogenomic treatment of insects^27^, one main difference is the position of Zoraptera. At least ten competing hypotheses have been proposed regarding the phylogenetic position of this order during the last century^42^. Molecular evidence accumulated during the past decade has convincingly shown that Zoraptera belongs to Polyneoptera, and accordingly the number of competing hypotheses was reduced to three (Table 2). Of these three alternatives, the sister group relationship between Zoraptera and Embiodea inferred from mitochondrial genomes^43^ was likely caused by long-branch attraction, because both lineages exhibit a significantly accelerated substitution rate in comparison with other polyneopteran orders^43^,^44^. The sister relationship between Zoraptera and Dermaptera was supported by 77% bootstrap values in the ML analysis of phylogenomic data based on supermatrix C^27^, which took amino acids as data type with 479 protein domain-based meta-partitions. Although only the chronogram based on supermatrix C was mentioned in that paper, the results based on a supermatrix D (a “good” site sub-alignment of supermatrix C) and four-cluster likelihood mapping (FcLM) corresponding to both supermatrices were provided as supplementary materials. In the phylogenetic result based on supermatrix D, the bootstrap value for the clade Zoraptera + Dermaptera was down to 50%. The results of FcLM together with permutation tests indicate that the majority of the likelihood for a Zoraptera + Dermaptera clade based on supermatrix C, and about half of that based on supermatrix D can be attributed to noise. The sister-group relationship between Zoraptera and Dermaptera inferred from transcriptomic data was therefore likely a false positive. However, molecular synapomorphies supporting a Zoraptera + Dictyoptera clade were documented by Wang *et al*.^32^. The present study provides further support to this hypothesis. Zoraptera have retained several plesiomorphic characters belonging to the groundplan of Neoptera^45^, and it is therefore difficult to resolve the order’s phylogenetic placement based on morphological characters. A sister-group relationship between Zoraptera and Dictyoptera was proposed by several authors (reviewed by Mashimoto *et al*.^42^). Although no morphological data seems to seriously conflict with this hypothesis, it cannot be considered conclusive as there are few synapomorphies supporting the node.

Another significant difference between our results and the phylogenomic study of insect^27^ is the monophyly of Paraneoptera. Our phylogenetic results support the monophyly of Paraneoptera (see Fig. 2 and Supplementary Fig. S1), which is consistent with the evidence both from morphological characters^38^,^46^,^47^, and molecular studies^28^,^48^. Conflicting with the above mentioned result of Misof *et al*.^27^, at least five non-homoplasious apomorphies support the monophyly of Paraneoptera, i.e., an elongate and stylet-like lacinia detached from stipes, an enlargement of clypeus and its associated muscles that are inserted on the dorsal wall of the preoral chamber, an anterior shift of the abdominal ganglia and their fusion with the metathoracic ganglia, the inflated anterior region of the second axillary sclerite, and the unique forewing venation^1^,^38^,^47^,^49^,^50^. There is no convincing morphological evidence for Psocodea + Holometabola. Furthermore, in the results of FcLM analyses together with permutation tests based on both supermatrices C and D, the likelihood of a clade formed by Psocodea + Holometabola was not favored if compared to those of competing hypotheses. Indeed, this phylogenetic position for Psocodea was mainly contributed by noise in the data^27^.

For the deeper divergences of insects, we explored the impact of different settings of SD values and different prior distributions on time estimates. Firstly, we adopted different settings of the SD for the normal distribution in the UCL clock model. The results show that the differences for most nodes are relatively small under various settings (Table 1). Secondly, the impact of different prior distributions on divergence times were evaluated. Compared to the analysis implemented in BEAST^51^ using normal prior distributions, the median ages of divergence times using uniform prior distributions implemented in MrBayes^52^ showed only a few differences within Neoptera except of the supercohort Polyneoptera. For the nodes Dicondylia, Palaeoptera, and Pterygota, the estimated divergence times were relatively close to each other under two different prior distributions. While for the remaining two nodes, i.e., root, and Ectognatha/Insecta, the estimated divergence times with uniform prior distributions were underestimated to a large extent. Such postponement is probably due to the limitation of the available fossils of Palaeoptera selected as calibration points. Although the present study involved some of the earliest presently known fossils, it is likely that even earlier fossils are yet to be discovered.

We further assessed whether the use of priors has an influence on the posterior estimates for the three groups of SD values by comparing our data driven posterior estimates with the results of prior-only analyses based solely upon fossil priors (i.e., without molecular data). Among the calibration points, the posterior distributions of age estimation were older than the prior distributions in case of four nodes (Dicondylia, Notoptera, Psocodea, and root), younger in another four nodes (Diptera, Neuropterida, Ephemeroptera, and Odonata), and approximately the same in the remaining eight nodes (Supplementary Table S1). As for the prior settings with increased SD values, the posterior distribution and the estimated divergence time for deeper nodes did not change substantially, therefore the calibrations are not overly informative on the posterior distribution. Our data did influence our posterior estimates significantly, suggesting that our soft priors did not cause overparameterization and thus they are biased to our posterior estimates^19^.

Our inferred divergence times suggest that stem-group insects diverged from their common ancestor with the more basal (entognathous) hexapods around the Early Ordovician, approximately 475 Ma. This result is slightly younger than the phylogenomic study of Misof *et al*.^27^ which dated the origin of Hexapoda to 493 Ma. In either case, this is long before the origin of terrestrial animal life^53^,^54^ and thus the early lineages of Hexapoda, basal to Entognatha + Insecta, most likely were marine. It remains unknown when hexapods transitioned to land, but it is likely that besides arachnids and myriapods, ancient hexapods were also present in the complex terrestrial ecosystems already existing in the latest Silurian^2^,^53^. These data stress a definite need to scour marine deposits for stem-group hexapods.

Following the colonization of land by hexapods in the Silurian, insects evolved flight. Interestingly, the origin of the winged insects (Pterygota) is dated at about 413 Ma, only slightly prior to the age of *Rhyniognatha hirsti*. These results indicate that the acquisition of wings did not take long once hexapods transitioned to land and diverged into entognathous hexapods and true insects. Our estimate of the earliest diversification of the winged insects is in line with the evidence obtained from a larger dataset^27^. This correlated evidence for the time of origin for powered flight is critical for correctly placing wing origins into a proper paleoecological context^55^, and further points toward a terrestrial origin for pterygotes, with subsequent invasions of freshwater habitats independently among the paleopterous orders (Ephemeroptera and Odonata), and subsequently among subsets of the Neoptera, particularly because freshwater habitats were not abundant in the earliest Devonian. It also correlates with a period ca. 408 Ma characterized by a hyperoxic atmosphere. As metabolically intensive flight muscles demand oxygen at a fast rate, an oxygen concentration as high as approximately 24–25% greatly facilitated acquisition of flight^56^.

Subsequently, waves of diversification of winged insects apparently correlate with major changes in terrestrial floras. The establishment of terrestrial floras during the Devonian can largely be divided into three main stages, i.e., the short, riparian rhyniopsid-dominated habitats in the earliest Devonian, the arborescence plants evolving period from the latest Pragian through the Givetian (412–370 Ma), and the emerging of medium-sized to giant tree fern forests (e.g., cladoxylopsids, lycopsids) in the Late Devonian, which also witnessed the appearance of the first seed plants near the end of the Devonian (Famennian, ca. 364 Ma). Following the serial colonization and radiation of vascular plants across the Devonian and seed plants in the Early Carboniferous, there appear to be three important events in the evolutionary history of insects, i.e., an earliest Devonian origin of flight, a late Emsian to Famennian set of cladogenetic events giving rise to the stem paleopterous, neopterous, and eumetabolan insects, and an late Devonian to Mississippian event marking the radiation of paraneopteran, holometabolan, and polyneopteran evolution (Fig. 3).

Insects would have benefited substantially from the nutritious resources and the structurally heterogenous niches offered by these new plant lineages. It is perhaps not surprising that a significant portion of paraneopteran and holometabolan diversification among the ordinal lineages is reflected in mouthpart specializations, following the prior dramatic changes in the available floral food resources. Indeed, the groundplans for many clades, including early saprophagous, mycophagous, predacious, and omnivorous lineages, reflect an increased variety in diet. Taking Paraneoptera for example, its diversification timescale is congruent with the time of the origin and initial diversification of seed plants^1^, which probably promoted the evolution of the mouthparts within the supercohort. During this time, paraneopteran mouthparts experienced two evolutionary changes, i.e., from the “chewing” mouthparts of Psocoptera to the probing and puncturing mouthparts present in stem Condylognatha^57^, then to the distinctive piercing-sucking rostrum or beak with suppressed mandibular and maxillary palps in the Hemiptera. Besides, spatial heterogeneity was also altered significantly during the latter half of the Devonian, with the increasing height and diversity of sciophilous plants concealed spaces would have become more varied and numerous. Among polyneopterans and paraneopterans a preference for cryptic habitats is frequent.

**Figure 1:**
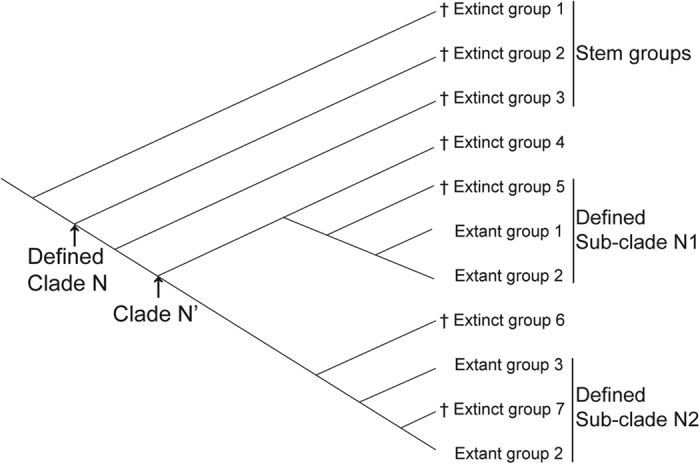
Possible positions for the fossil record in a defined clade. A crisscross “†” stands for the fossil record of the corresponding group.

**Figure 2:**
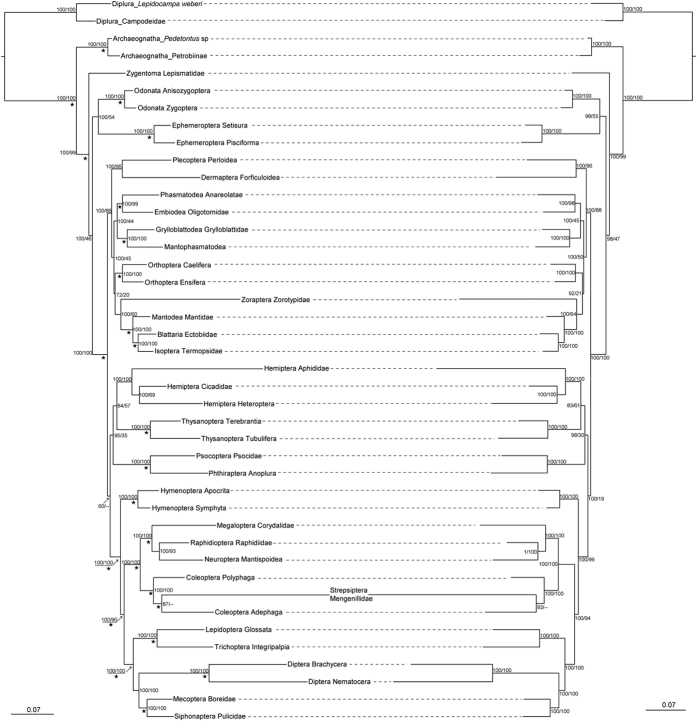
Phylograms inferred from the nuclear genes. The left tree is based on the five nuclear genes with DNA model applied to the paired sites of rDNAs, while the right tree is based on the five nuclear genes with Doublet model applied to the paired sites of rRNAs. Numbers associated with each node indicate Bayesian posterior probabilities values and maximum-likelihood (ML) bootstrap values. An asterisk denotes that the clade is also present in the maximum parsimony analysis (only values ≥60% are shown). A dash is shown if the topology is not shown in the maximum likelihood analysis. The lengths of the branches follow the phylograms of the BI trees.

In this study, we preliminarily explored the application of stem-group fossils as independent evidence in divergence time estimations. As shown in Fig. 1, for a defined clade N, the inferred divergence time through the node-dating method is actually the time of clade N’, and usually there is a time interval between them. According to the diversification of winged insects in the Carboniferous, all of the currently earliest fossils of stem groups in Neoptera fall in the distant direction of the inferred divergence times.

Our results suggest that the radiation of Polyneoptera occurred in the Early Carboniferous, which is about 53 million years earlier than the time inferred from phylogenomic data^27^. Within Polyneoptera, the divergence time between Notoptera and Eukinolabia is approximately 50 million years older than that inferred from phylogenomic data^27^. The radiation of Blattopterida (=Zoraptera + Dictyoptera) is estimated to begin at 273 Ma; this event was not recovered by Misof *et al*.^27^. Without the recognition of a relationship between Zoraptera and Dictyoptera, the discrepancies between the divergence times indicated by stem-group fossils and that inferred based on molecular data can be at least 110 million years, which throws some concern over estimates not incorporating such evidence. Considering a monophyletic Zoraptera + Dictyoptera, the estimated divergence time for Blattopterida, is about 40 million years later than the estimation indicated by putative stem groups.

**Figure 3:**
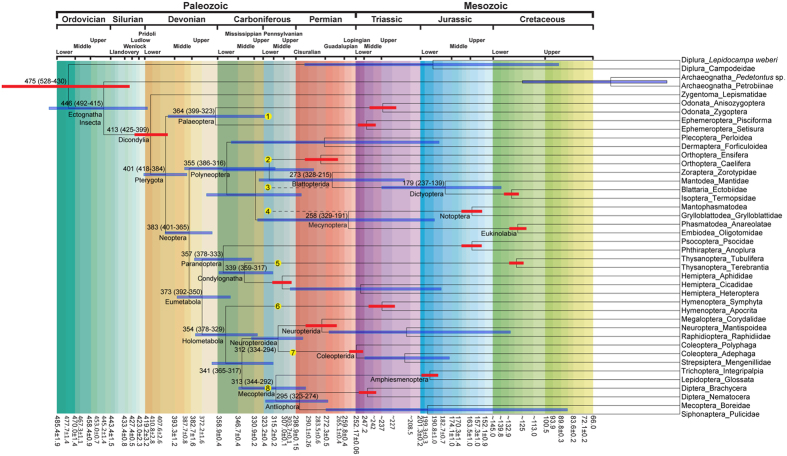
The maximum clade credibility chronogram from BEAST analysis. The blue bars illustrate the extent of the 95% highest posterior density credibility intervals for each divergence time. The detailed estimated time for the deep divergences of winged insects were provided. The numbers on yellow solid circle background indicates the first appearances of the stem groups for certain clades, while the nodes with orange 95% HPD bars indicated the calibrated points. Numbers from one to eight are stand for species *Delitzschala bitterfeldensis*, Archaeorthoptera, *Qilianiblatta namurensis, Sinonamuropteris ningxiaensis, Westphalothripides oudardi, Avioxyela gallica, Stephanastus polinae*, and *Westphalomerope maryvonneae* respectively.

Despite this, stem-group fossils belonging to Dictyoptera and Notoptera, most notably *Qilianiblatta namurensis* and *Sinonamuropteris ningxiaensis* from the Bashkirian (323.2–315.2 Ma) (circles 2, 3, and 4 in Fig. 3 and Supplementary Table S2), indicate that the inferred divergence times within Polyneoptera are still underestimated. In fact, the divergence times within Polyneoptera inferred by uniform prior distributions are approximately 20 to 40 million years earlier than the results inferred by normal prior distributions. It is likely that early radiations have happened during the Mississippian in each of the three supercohorts (Polyneoptera, Paraneoptera, and Holometabola). The best explanation for the gaps between the times of the fossil record of stem groups and that of the molecular-based estimation is probably the distant positions of the stem groups relative to their crown-group counterparts (Fig. 1). During evolution a given stem group can give rise to an independent clade before the diversification of the order and it does not belong to any extant order (extinct group 1 in Fig. 1). However, the node-dating method can estimate only the divergence times of the extant groups. Therefore, the results based on the clock model arguably can underestimate divergence times for some nodes.

In Holometabola, most of the time gaps between stem-group fossils and molecular-based estimations were remarkably narrow. The available stem-group fossils of Mecopterida (circle 8 in Fig. 3 and Supplementary Table S2) fit into the distant part of the estimated 95% HPD. The diversifications of the remaining hyperdiverse holometabolan clades are also in accordance with recent paleontological discoveries from the Carboniferous^4^, such as the earliest fossils of stem-group Coleopterida (circle 7 in Fig. 3 and Supplementary Table S2). Taking into consideration the co-evolutionary relationships between holometabolous insects and plants it is unlikely that Holometabola had a diversification much earlier than the radiation of seed plants. The estimated divergence times of holometabolans in this study are close to the age of the available stem-group fossils, and thus permit a rather robust perspective on early insect evolution. The estimated divergence times of the main holometabolan clades inferred from phylogenomic data^27^ are usually younger than the corresponding times inferred in the present study, except for the divergence time between Neuropterida and Coleopterida which is almost the same. The selection of younger fossils can be one of the reasons for the younger times inferred in the work of Misof *et al*.^27^.

The chronostratic time of the fossil record of stem groups of Palaeoptera is later than the inferred divergence time based on molecular data (circle 1 in Fig. 3 and Supplementary Table S2). This discrepancy apparently can be resolved by two considerations. First, relevant well-preserved fossils are largely unavailable due to the paucity of deposits in the Devonian and Mississippian. Second, the phylogenetic context has not become stable enough to summarize morphological apomorphies until a more recenttly^27^,^30^,^31^,^32^. In the absence of convincing apomorphies for the supercohorts of extant orders it is difficult to assign certain fossils to stem groups, and there is a considerable disagreement among paleontologists about the taxonomic placement of a number of these taxa. Because the current view on the phylogenetic relationships of insects at the ordinal level is considered as satisfyingly accurate except for a few controversial nodes, this background provides more opportunities for determination of the systematic positions of several problematic stem groups, and also facilitates a better recognition of the synapomorphies of most of the higher-level insect clades. The gaps between the chronostratic times of the stem groups and the inferred divergence times based on molecular data will most likely be further narrowed in the future.

The revised chronogram for the phases of early insect diversification correlate with paleoecological and paleoclimatological shifts, and help to clarify in which geological stages particular cladogenetic events took place. The present study is the first to demonstrate the advantage of a combined employment of stem group fossils and molecular markers in studies of divergence time estimation. The inferred divergence times for the radiations within the major linages of Neoptera in the Carboniferous were largely in congruence with the earlier available fossils of certain stem groups. This approach can be utilized in chronological studies on other groups, especially those that have limited co-evolutionary relationships.

## Methods

### Taxon sampling, sequence alignment, and dataset concatenation

A dataset comprising 5 nuclear genes and 13 mitochondrial PCGs from 42 representatives of all 30 insect orders was compiled (Supplementary Table S3). Two representatives of Diplura were used as outgroups.

All molecular sequences were pre-aligned using Muscle embedded within MEGA 6^58^,^59^. For rDNAs, the automatic alignment results were then checked and corrected manually referring to the consensus secondary structure models of insects’ 18S and 28S rRNAs respectively^32^,^60^. In both of these two previous works, the secondary structures especially for the hyper-variable regions were refined by re-calculation with RNAstructure 5.6^61^ and comparative methods to make them more suitable for insects. Compensatory or semi-compensatory substitution can help to verify the paired regions. More details for manual alignment have been mentioned repeatedly in the previous studies^28^,^32^. After this process, ambiguously aligned positions for rDNAs were manually excluded prior to the phylogenetic reconstruction. For the three nuclear PCGs, the length variations of them are not as large as those of the rDNAs between different insect orders. Before the phylogenetic reconstruction, we only removed the columns which present in no more than 70% sampled groups in both ends. The amino acid sequences of corresponding 13 mitochondrial PCGs were aligned firstly in codon-based mode and then toggled back to the nucleotide sequences. The columns with taxon coverage lower than 70% in both ends were removed as well before the phylogenetic reconstruction. Saturation test for each of the three codon positions of mitochondrial PCGs was assessed by DAMBE 5^62^.

Alignments of individual genes were concatenated as five matrices. The first matrix, which serves as a basic one (matrix 1), was composed of nucleotide sequences of two rDNAs and amino acid sequences of three nuclear PCGs. Based on this basic matrix, the remaining four matrices (matrices 2–5) were built via plus the first codon positions (PCG1), the second codon positions (PCG2), the first two codon positions (PCG12), and the amino acid sequences (PCGAA) of the 13 mitochondrial PCGs respectively.

### Phylogenetic reconstruction

Phylogenetic analyses were performed by using BI as implemented in MrBayes 3.2.5^52^, and ML as implemented in RAxML 8.0.12^63^, and MP as implemented in TNT^64^. The programs jModeltest 2.1.1^65^ and Treefinder (http://www.treefinder.de/) were used to infer the best substitution model for the nucleotides and amino acids, respectively. The parameters in jModeltest are set as follows. The number of substitution schemes equaled to 11, the base tree for likelihood calculations was ML optimized, and the base tree search was best. Whether under the Bayesian Information Criterion (BIC) or the corrected Akaike Information Criteria (AICc), GTR was shown to be the best-fitting model for the rDNA sequences, and the PCG1 and PCG2 of mitochondrial PCGs. While the best nucleotide substitution model for the PCG12 of mitochondrial PCGs was TVM. All sets in Treefinder analysis were left to the default values. The most appropriate substitution model for the amino acid sequences was LG for proteins DPD1 and ATP8, and JTT for RPB1, RPB2, and ND3 under both of the two criteria as well. For the amino acid sequences of proteins ATP6, CO1, CO2, CO3, and ND1, the best-fit substitution model was MtArt. For the amino acid sequences of the remaining 6 proteins, i.e., Cytb, ND2, ND4, ND4L, ND5, and ND6, the best-fit one was mtZOA. All of the substitution models included a four category discrete approximation to a gamma distribution (+G) with a proportion of invariable sites (+I) to account for among-site rate variation.

For MrBayes, other parameters were set as follows: generations = 5,000,000, samplefreq = 100, nchains = 4, nst = 6. Convergence was assessed by the SD values and also by the trace plot and effective sample size (ESS) values in Tracer v1.5 (http://beast.bio.ed.ac.uk/Tracer). When the average standard deviations of split frequencies fell below 0.01, the generations with corresponding values higher than 0.01 were discarded as burn-in and the remaining sampled trees were used to estimate posterior parameters and probability distributions. For RAxML, the best ML tree was calculated from 200 RAxML runs, followed by 1,000 bootstrap replicates. For the rDNA sequences in the basic matrix, additional phylogenetic reconstructions employing an RNA model (doublet model) for the stem-encoding regions was conducted with MrBayes and RAxML. And the remaining parameters are the same as in the aforementioned phylogenetic analyses.

### Divergence time estimation

As the phylogenetic reconstructions including mitochondrial PCGs in the datasets could not reach consistence between different algorithms (see the section of results), divergence times were estimated based on the dataset composed only by the nuclear genes (matrix 1). BEAST 2.4.1^51^ was used to estimate divergence times following the topology obtained in the phylogenetic reconstructions. Data were partitioned into three parts. The first part is the rDNA sequences, the rest two parts are the amino acid sequences. The substitution models for rDNAs and amino acids were applied as the same as those applied in the RAxML analysis. The Birth-Death model of speciation^66^ and an uncorrelated log-normal relaxed clock^67^ were employed. Because the monophyly of Eumetabola has received support from various kinds of evidence such as the morphology, rDNAs, ESTs, and transcriptome data^27^,^32^,^39^,^47^,^48^, the node Eumetabola was also included in the fixed topology in the analyses of divergence time estimation. All other priors, except the calibration points described below, were left to the defaults in BEAST.

The analyses using BEAST were run for a total of 100,000,000 generations and were sampled every 100 generations. The program Tracer v1.6 (http://beast.bio.ed.ac.uk/Tracer) was used to examine the posterior distribution of all parameters and their associated statistics, such as the ESS and the 95% HPD intervals. The program TreeAnnotator v2.4.1^51^ was used to summarize the set of post burn-in trees and their parameters, to produce a maximum clade credibility chronogram that showed mean divergence time estimates with 95% HPD intervals. All of the ESSs were above the recommended threshold of 200, which indicates that the parameter space had been sufficiently sampled. The chronological coordinate in the chronogram is referenced to the International Chronostratigraphic Chart^68^.

The earliest fossils for each taxon were extracted from the fossil insect database Paleobiology Database (http://paleobiodb.org/cgi-bin/bridge.pl) and EDNA (http://edna.palass-hosting.org/search.php). Furthermore, these fossils have been verified according to corresponding references (Supplementary Table S4). In order to have comprehensive representatives and balanced calibrations in each major supercohort of the tree, analyses were based on 16 calibration points in various lineages. All calibration points were set as normal distribution. As the possibility for overestimation of the fossil record cannot be completely eliminated^18^,^69^, the normal distribution was used to reflect potential uncertainty in the fossil record and allows the posterior estimate to vary in either direction^69^. Each fossil selected for time calibration is the earliest one belonging to one of the sister groups (extant taxa) according to a certain node. The fossil calibrations were set as follows. i) The oldest fossil record of Dicondylia, i.e., *Rhyniognatha hirsti*, was used to calibrate the node Insecta. ii) the fossil *Triassonurus doliiformis* assigned to the extant family Siphlonuridae from the Middle Triassic was used to calibrate the node Ephemeroptera. iii) *Triassothemis mendozensis* of the fossil family Triassolestidae (crown group of Epiprocta, comprising Anisozygoptera and Anisoptera), was used to calibrate the node Odonata. iv) *Raphogla rubra*, which is thought to be the oldest representative of modern Ensifera, was used to calibrate the split between the two suborders Ensifera and Caelifera. v) *Juramantophasma sinica*, which is likely a stem lineage of Mantophasmatodea, was used to calibrate the node Notoptera. vi) *Cretophasmomima melanogramma*, which can be definitely assigned to the stem lineage of Phasmatodea, was used to calibrate the split between Phasmatodea and Embiodea. vii) *Valditermes brenanae*, a stem lineage of Mastotermitidae, was used to calibrate the node Blattodea. viii) One Early Cretaceous fossil *Tethysthrips libanicus* of Thripidae was used to calibrate the split between the two suborders Tubulifera and Terebrantia of Thysanoptera. ix) *Aviorrhyncha magnifica*, the stem lineage of Euhemiptera (containing all living Hemiptera except Sternorrhyncha), was used to calibrate the split between Sternorrhyncha and Euhemiptera. x) *Paramesopsocus adibi* (Psocodea: Psocoptera: Troctomorpha: Electrentomidae) was used to calibrate the node Psocodea (comprising Psocoptera and Phthiraptera). xi) *Triassoxyela foveolata*, a stem group of the family Xyelidae, was used to calibrate the split between Archihymenoptera and Neohymenoptera. xii) *Elmothone martynovae*, a stem lineage of Neuroptera, was used to calibrate the Neuropterida. xiii) *Triaplus sibiricus*, which belongs to the fossil family Triaplidae (Coleoptera: Adephaga), was used to calibrate the node Coleopterida. xiv) *Archaeolepis mane* from the Early Jurassic, which is thought to be a stem lepidopteran, was used to calibrate the split between Lepidoptera and Trichoptera. xv) *Archilimonia vogesiana* (Diptura: Tipulomorpha: Pediciidae) was used to calibrate the node Diptera. The fossil record *R. hirsti* was also used to calibrate the root but with a soft maximum constraint. The maximum bound of the 95% confidence interval (CI) was established on the age of the *Yicaris dianensis* (the oldest fossil record of Arthropoda), the age of which has been dated to 521 Ma. We also provide explicit descriptions of each fossil record, especially phylogenetic justifications and age justifications (Table 3 and Supplementary File S5).

In addition, the impact of different SD values on age estimates were assessed. The first one is to set the mean to the middle value of the Stage/Age in which the corresponding fossil located, and use the starting time of additional one earlier Stage/Age and the ending time of additional one later Stage/Age as the bounds for the 95% CI. The second one is to reduce the range of 95% CI and use the starting and ending time of the Stage/Age where the fossil locates as the bounds. The third one is to expand the 95% CI and use the starting time of additional two earlier Stages/Ages and the ending time of additional two later Stages/Ages as the bounds. The prior distributions for them were tabulated for easy access (Supplementary Table S4).

We also tested the effects of using uniform prior distributions instead of normal distribution on age estimates. The influence of secondary structure on divergence time estimation was assessed as well. These analyses were carried out with MrBayes 3.2.5^52^. All substitution model parameter settings were the same as those used in the phylogenetic analysis by Bayesian inference. Such analyses were run 10,000,000 generations, sampling every 100 generations. For the relaxed clock, the independent gamma rate (IGR) model^70^ was employed, under a uniform branch length prior. All calibration priors were set as uniform distributions, with the upper bound in which the corresponding fossil located as minimum bounds. For the nodes Odonata and Ephemeroptera, the maximum bounds were set as 521 Ma, while for remain nodes the maximum bounds were set as 411 Ma according to the time of the oldest fossil record of Hexapoda.

### Ethical statement

The species used in this study are not included in the “List of Protected Animals in China”. No specific permits were required for collecting insects from the Experimental Animal Ethics Committee of Nankai University. The field studies did not involve endangered or protected species.

## Additional Information

**How to cite this article**: Wang, Y.-h. *et al*. Fossil record of stem groups employed in evaluating the chronogram of insects (Arthropoda: Hexapoda). *Sci. Rep.* **6**, 38939; doi: 10.1038/srep38939 (2016).

**Publisher’s note:** Springer Nature remains neutral with regard to jurisdictional claims in published maps and institutional affiliations.

## Supplementary Material

Supplementary Materials

## Figures and Tables

**Table 1:** Divergence times of the deeper nodes with alternative prior settings and different clock models.

Divergence times of the deeper nodes	BEAST analyses (normal) (mean age and the 95% HPD, Ma)			Bayesian analyses (uniform) (mean age and the 95% HPD, Ma)	
	±one Stage/Age	Within certain Stage/Age	±two Stage/Age	RNA models	DNA models
	UCL	UCL	UCL	IGR	IGR
Root	475 (430–528)	468 (430–508)	475 (437–521)	410 (406–415)	410 (406–415)
Ectognatha/Insecta	446 (415–492)	442 (418–480)	448 (421–483)	408 (406–412)	408 (406–412)
Dicondylia	413 (399–425)	409 (404–414)	413 (398–428)	406 (405–409)	406 (405–409)
Palaeoptera	364 (323–399)	353 (301–385)	357 (313–395)	363 (310–399)	367 (322–398)
Pterygota	401 (384–418)	398 (384–408)	401 (380–418)	402 (391–408)	401 (392–408)
Neoptera	383 (365–401)	378 (358–393)	379 (357–400)	395 (382–404)	394 (381–404)
Polyneoptera	355 (316–386)	349 (319–381)	343 (308–373)	374 (344–397)	374 (345–396)
Dictyoptera	179 (139–237)	173 (141–236)	179 (138–234)	205 (151–266)	205 (143–263)
Mecynoptera	258 (191–329)	256 (186–328)	265 (203–326)	305 (241–358)	300 (240–354)
Eumetabola	373 (350–392)	368 (350–385)	369 (348–390)	386 (370–401)	386 (367–400)
Paraneoptera	357 (333–378)	353 (333–372)	353 (329–377)	369 (344–393)	369 (343–390)
Condylognatha	339 (317–359)	336 (320–356)	335 (315–360)	349 (322–374)	348 (319–375)
Holometabola	354 (329–378)	347 (327–368)	350 (331–371)	373 (349–394)	371 (349–392)
Neuropteroidea	312 (294–334)	306 (288–328)	308 (283–332)	332 (290–364)	332 (301–362)
Mecopterida	313 (292–344)	312 (292–338)	315 (293–339)	337 (299–370)	335 (302–367)
Neuropteroidea + Mecopterida	341 (317–365)	334 (316–356)	339 (318–360)	360 (334–386)	359 (330–382)
Antliophora	295 (274–323)	292 (273–319)	295 (276–314)	308 (270–358)	308 (268–354)

**Table 2:** Three main competing hypotheses for sister group relationship of Zoraptera within Polyneoptera based on molecular data.

Hypotheses	Data type	Possible reason of false positive result
Zoraptera + Embiodea	mitochondrial genomes	Long branch attraction
Zoraptera + Dictyoptera	five nuclear genes	—
Zoraptera + Dermaptera	transcriptome data	noises

**Table 3:** Fossil Record used in the analyses for estimating divergence times.

Taxonomic group	Fossil taxon	Locality	Mode of Preservation	Age (Ma)
stem Lepidoptera	*Archaeolepis mane*	Black Ven, Charmouth, Dorset, England	compression fossil	Sinemurian (199.3 ± 0.3–190.8 ± 1.0)
Blattodea - Mastotermitidae	*Valditermes brenanae*	Clockhouse Brickworks	impression	Hauterivian (132.9–129.4)
Coleoptera - Adephaga	*Triaplus sibiricus*	Babii Kamen’	impression	Changhsingian-Induan (254.14 ± 0.07–251.2)
Dicondylia	*Rhyniognatha hirsti*	Rhynie cherts	chert	Pragian (410.8 ± 2.8–407.6 ± 2.6)
Diptera - Tipulomorpha - Pediciidae	*Archilimonia vogesiana*	Bust, VosgesMts, France	impression	Anisian 247.2–242.0
stem Phasmatodea	*Cretophasmomima melanogramma*	Liutiaogou Village, Dashuangmiao Town	compression fossil	Barremian-Early Aptian (129.7 ± 0.5–122.1 ± 0.3)
Ephemeroptera- Siphlonuridae	*Triassonurus doliiformis*	Arzviller	impression	Anisian (247.2–242.0)
stem Euhemiptera (=Hemiptera except Sternorrhyncha)	*Aviorrhyncha magnifica*	Terril No 7, Avion	impression	Moscovian (315.2 ± 0.2–307.0 ± 0.1)
Hymenoptera - Xyelidae	*Triassoxyela foveolata*	Madygen, Kyrgyzstan	compression fossil	Carnian (237.0–227.0)
stem Neuroptera	*Elmothone martynovae*	Elmo, MCZ 1927 collection	impression	Artinskian – Kungurian (290.1 ± 0.26–272.3 ± 0.5)
Odonata-Epiprocta (=Anisozygoptera + Anisoptera)	*Triassothemis mendozensis*	Agua de las Avispas	compression fossil	Carnian (237.0–227.0)
Orthoptera - Ensifera	*Raphogla rubra*	F21D, Le Moural D, Lodève Basin	impression	Artinskian- Kungurian (290.1 ± 0.26–272.3 ± 0.5)
Psocoptera - Troctomorpha - Electrentomidae	*Paramesopsocus adibi*	Karatau-Mikhailovka	cast	Callovian – Oxfordian (166.1 ± 1.2–157.3 ± 1.0)
Thysanoptera - Thripidae	*Tethysthrips libanicus*	Mdeyrij-Hammana, Casa Baabda	amber	Barremian (129.4–125.0)
stem Mantophasmatodea	*Juramantophasma sinica*	Daohugou	compression fossil	Callovian – Oxfordian (166.1 ± 1.2–157.3 ± 1.0)
