# Supplementary material for: Fossil record of stem groups employed in evaluating the chronogram of insects (Arthropoda: Hexapoda)
Source: Sci Rep. 2016 Dec 13;6:38939. doi: 10.1038/srep38939 (PMC5154178; doi:10.1038/srep38939)
Supplement: Supplementary Materials [file srep38939-s1.pdf]

# Supplementary Materials for

# Fossil record of stem groups employed in evaluating the chronogram of insects (Arthropoda: Hexapoda)

Yan-hui Wang[1,2,†], Michael S. Engel[3,†], José A. Rafael[4,†], Hao-yang Wu[2], Dávid Rédei[2], Qiang Xie[2,*], Gang Wang[1], Xiao-guang Liu[1] and Wen-jun Bu[2]

## Supplementary material legends:

**Table S1. Comparison between the prior and posterior marginal densities of calibrated points under various settings of the lower and upper bounds of 95% HPD in normal distribution.** The blue indicates the prior distribution and the gray indicates the posterior distribution.

**Table S2. Stem group fossils collected from the fossil insect database Paleobiology Database and EDNA**.

**Table S3. Taxon sampling of nuclear genes and mitochondrial genomes.** The sequence indicated by asterisk was newly sequenced in this study.

**Table S4. Fossil record and prior distributions for different analyses**.

**File S5. Detailed phylogenetic justification and age justification for each fossil item.**

**Fig. S1. Phylogenetic trees inferred from matrix 1, i.e., the combined dataset of rDNAs and protein-coding genes.** A) ML topology with DNA model applied to the paired sites of rDNAs, B) MP topology, C) ML topology with Doublet model applied to the paired sites of rRNAs. Posterior probabilities and bootstrap values are shown at each node, respectively.

**Fig. S2. Phylogenetic trees inferred from matrix 2, i.e., the combined dataset of nuclear genes and the first codon positions of mitochondrial PCGs**. A) BI topology, B) ML topology, C) MP topology. Posterior probabilities and bootstrap values are shown at each node, respectively.

**Fig. S3. Phylogenetic trees inferred from matrix 3, i.e., the combined dataset of nuclear genes and the second codon positions of Mitochondrial PCGs**. A) BI topology, B) ML topology, C) MP topology. Posterior probabilities and bootstrap values are shown at each node, respectively.

**Fig. S4. Phylogenetic trees inferred from matrix 4, i.e., the combined dataset of nuclear genes and the first two codon positions of mitochondrial PCGs**. A) BI topology, B) ML topology, C) MP topology. Posterior probabilities and bootstrap values are shown at each node, respectively.

**Fig. S5. Phylogenetic trees inferred from matrix 5, i.e., the combined dataset of nuclear genes and the amino acid sequences of mitochondrial PCGs**. A) BI topology, B) ML topology, C) MP topology. Posterior probabilities and bootstrap values are shown at each node, respectively.

**Table S1. Comparison between the prior and posterior marginal densities of calibrated points under various settings of the lower and upper bounds of 95% HPD in normal distribution.** The blue indicates the prior distribution and the gray indicates the posterior distribution.

| Nodes | ± 1 Stage/Age | Within certain Stage/Age | ± 2 Stages/Ages |
|---|---|---|---|
| Amphiesmenoptera | 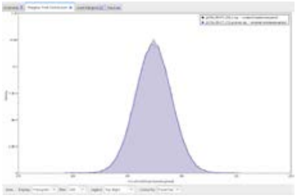 | 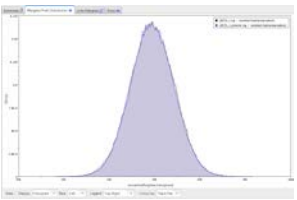 | 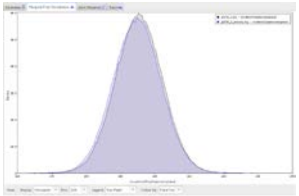 |
| Blattodea | 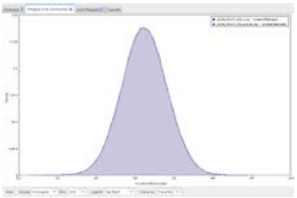 | 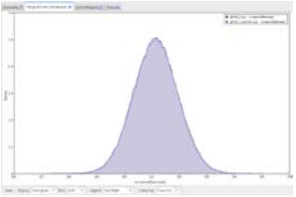 | 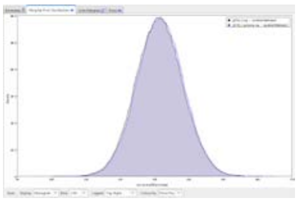 |
| Coleopterida | 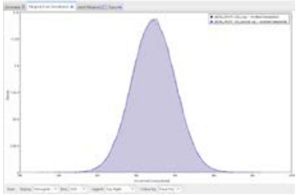 | 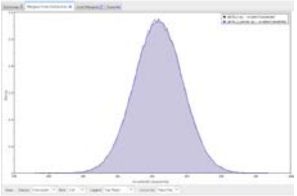 | 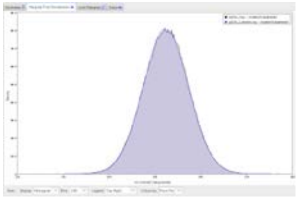 |
| Dicondylia | 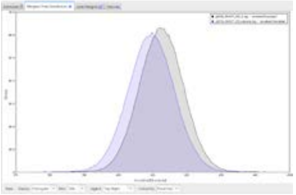 | 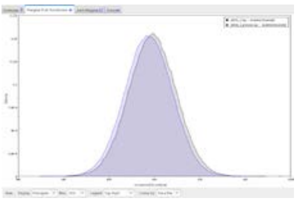 | 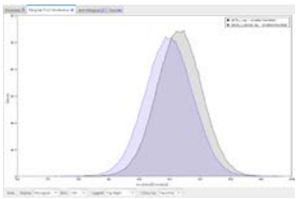 |
| Diptera | 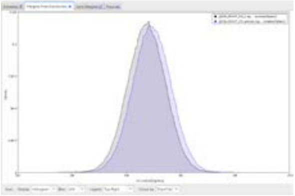 | 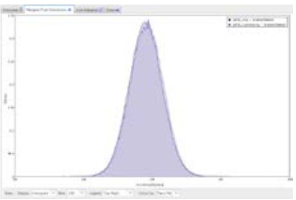 | 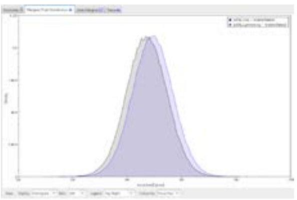 |
| Eukinolabia | 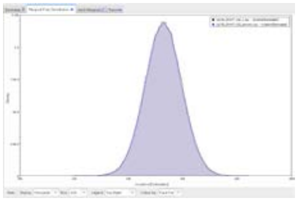 | 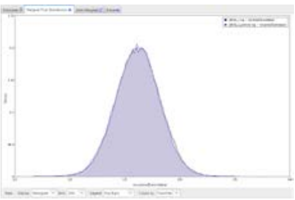 | 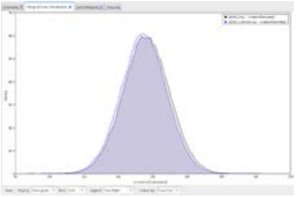 |
| Ephemeroptera | 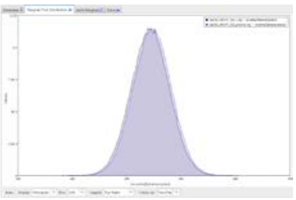 | 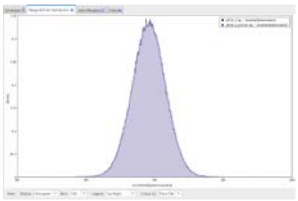 | 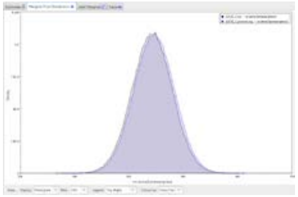 |

Hemiptera

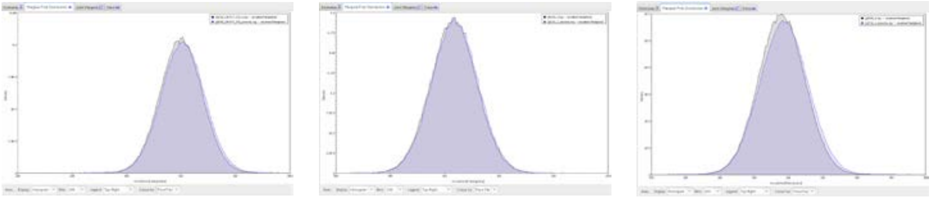

Hymenoptera

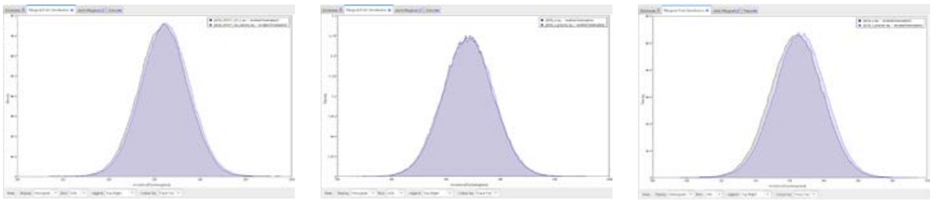

Neuropterida

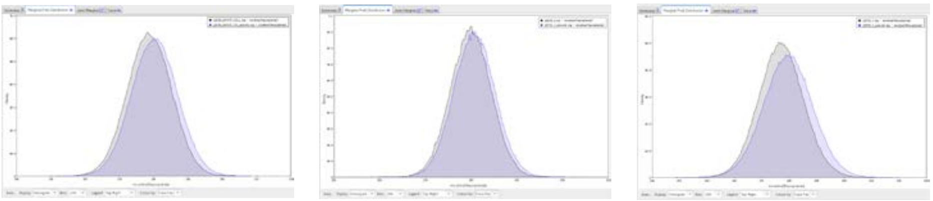

Odonata

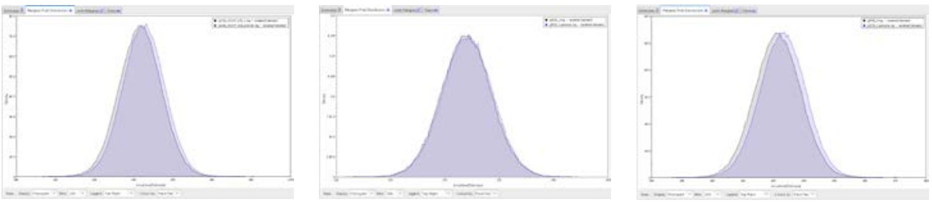

Orthoptera

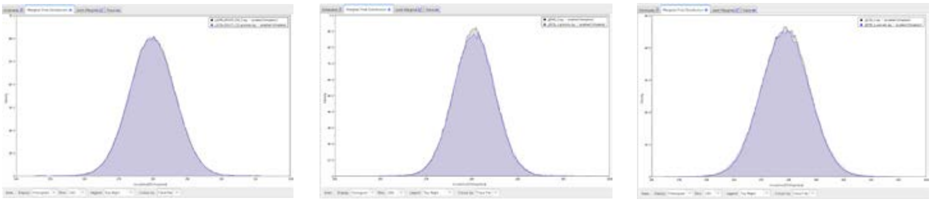

Psocodea

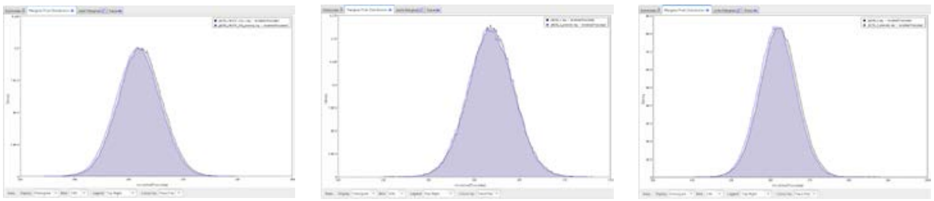

Thysanoptera

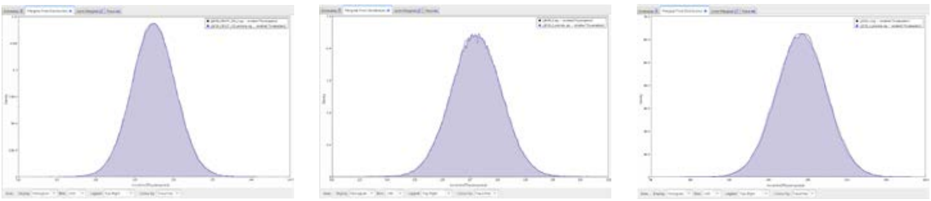

Notoptera

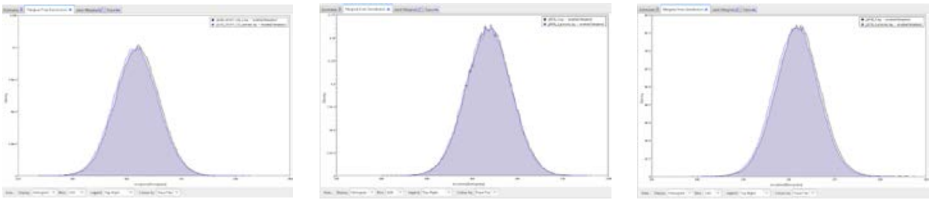

| | | | |
|---|---|---|---|
| Root | 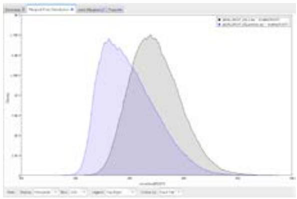 | 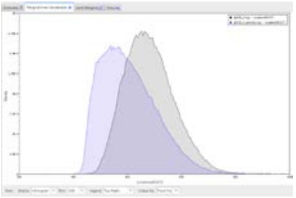 | 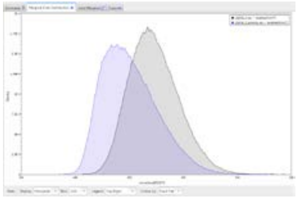 |

**Table S2. Stem group fossils collected from the fossil insect database Paleobiology Database and EDNA.**

| Nodes | Period | Age | Genus-species | References |
|---|---|---|---|---|
| Stem Palaeoptera | Carboniferous | Bashkirian 323.2-315.2 | *Delitzschala bitterfeldensis* | Brauckmann and Schneider 1996 |
| Stem Orthoptera | Carboniferous | Bashkirian 323.2-315.2 | Archaeorthoptera | Béthoux and Nel, 2002 |
| Stem Dictyoptera | Carboniferous | Bashkirian 323.2-315.2 | *Qilianiblatta namurensis* | Zhang et al. 2013 |
| Stem Notoptera | Carboniferous | Bashkirian 323.2-315.2 | *Sinonamuropteris ningxiaensis* | Peng et al. 2005 |
| Condylognatha - Thripida | Carboniferous | Moscovian 315.2-307 | *Westphalothripides oudardi* | Nel et al. 2012 |
| Stem Hymenoptera | Carboniferous | Moscovian 315.2-307 | *Avioxyela gallica* | Nel et al. 2013 |
| Stem Coleopterida | Carboniferous | Gzhelia 303.7-298.9 | *Stephanastus polinae* | Nel et al. 2013 |
| Mecopterida | Carboniferous | Bashkirian 323.2-315.2 | *Westphalomerope maryvonneae* | Nel et al. 2007 |

**Table S3. Taxon sampling of nuclear genes and mitochondrial genomes.** The sequence indicated by asterisk was newly sequenced in this study.

| | | Taxon of 28S rDNA | Acc. No. of 28S rDNA | Taxon of 18S rDNA | Acc. No. of 18S rDNA | Taxon of DPD, RPB1 & RPB2 | Acc. No. of DPD1 | Acc. No. of RPB1 | Acc. No. of RPB2 | Taxon of Mt-genomes | Acc. No. of Mt-genomes |
|---|---|---|---|---|---|---|---|---|---|---|---|
| Diplura | Campodeidae1 | *Lepidocampa weberi* | EU376050 | *Lepidocampa weberi* | AY037167 | *Lepidocampa weberi* | AB598693 | AB596892 | AB597583 | *Lepidocampa weberi* | NC_022675 |
| | Campodeidae2 | *Campodea augens* | EF199977 | *Campodea tillyardi* | AF173234 | *Metriocampa* sp. | AB598692 | AB596891 | AB597582 | *Campodea lubbocki* | NC_008234 |
| Archaeognatha | Petrobiinae1 | *Pedetontus okajimae* | EU376055 | *Pedetontus okajimae* | EU368614 | *Pedetontus unimaculatus* | AB598694 | AB596893 | AB597584 | *Pedetontus silvestrii* | NC_011717 |
| | Petrobiinae2 | Petrobiinae sp. NKU-010 | JQ309935 | Petrobiinae sp. NKU-010 | JQ309931 | *Petrobiellus takunagae* | AB598695 | AB596894 | AB597585 | *Petrobiellus sp.* | KJ754503 |
| Zygentoma | Lepismatidae | Lepismatidae sp. NKU-008 | JQ309933 | Lepismatidae sp. NKU-008 | JQ309928 | *Nipponatelura* sp. | AB598696 | AB596895 | AB597586 | *Atelura formicaria* | NC_011197 |
| Odonata | Anisozygoptera | *Epiophlebia superstes* | EU424328 | *Epiophlebia superstes* | AF461247 | *Epiophlebia superstes* | AB598699 | AB596898 | AB597589 | *Epiophlebia superstes* | JX050223 |
| | Zygoptera | *Rimanella arcana* | FJ009944 | *Devadatta podolestoides* | FN356078 | *Mnais pruinosa pruinosa* | AB598700 | AB596899 | AB597590 | *Vestalis melania* | NC_023233 |
| Ephemeroptera | Setisura | *Epeorus sylvicola* | EU414715 | *Macdunnoa hipawinia* | AY749874 | *Isonychia japonica* | AB598702 | AB596901 | AB597592 | *Isonychia ignota* | HM143892 |
| | Pisciforma | *Siphlonurus aestivalis* | EU414716 | *Edmundsius agilis* | AY749877 | *Ephemera japonica* | AB598701 | AB596900 | AB597591 | *Ephemera orientalis* | NC_012645 |
| Phasmatodea | Anareolatae | *Carausius morosus* | EU426878 | *Phobaeticus heusii* | AY121184 | *Phraortes illepidus* | AB598703 | AB596902 | AB597593 | *Phraortes illepidus* | NC_014695 |
| Embiodea | Oligotomidae | *Oligotoma* sp. NKU-001 | HQ857603 | *Oligotoma nigra* | Z97593 | *Aposthonia japonica* | AB598704 | AB596903 | AB597594 | *Aposthonia japonica* | AB639034 |
| Grylloblattodea | Grylloblattidae | *Galloisiana nipponensis* | DQ457320 | *Grylloblatta* sp. | AY521850 | *Galloisiana yuasai* | AB598705 | AB596904 | AB597595 | *Grylloblatta sculleni* | DQ241796 |
| Mantophasmatodea | Mantophasmatodea | *Tyrannophasma gladiator* | EU426875 | *Tyrannophasma gladiator* | AY521863 | *Karoophasma biedouwensis* | AB598706 | AB596905 | AB597596 | *Sclerophasma paresisense* | NC_007701 |
| Orthoptera | Caelifera | *Gomphocerinae* sp. JM-2004 | AY859546 | *Romalea* sp. | AY521868 | *Patanga japonica* | AB598707 | AB596906 | AB597597 | *Gomphocerus sibiricus* | NC_021103 |
| | Ensifera | *Leptophyes punctatissima* | EU414721 | *Pterophylla camellifolia* | AY121147 | *Euconocephalus varius* | AB598708 | AB596907 | AB597598 | *Conocephalus maculatus* | NC_016696 |
| Plecoptera | Perloidea | *Isoperla* sp. Plec1 | EU414717 | *Mesoperlina pecircai* | U68400 | *Oyamia lugubris* | AB598710 | AB596909 | AB597600 | *Kamimuria wangi* | NC_024033 |
| Dermaptera | Forficuloidea | *Forficula auricularia* | EU426876 | *Forficula* sp. NC-1995 | X89490 | *Forficula hiromasai* | AB598712 | AB596911 | AB597602 | *Challia fletcheri* | NC_018538 |
| Zoraptera | Zorotypidae | *Zorotypus weidneri* | JN192452 | *Zorotypus weidneri* | JQ259056 | *Zorotypus* sp. | AB598713 | AB596912 | AB597603 | *Zorotypus medoensis* | NC_026077 |
| Mantodea | Mantidae | *Mantis religiosa* | AY859585 | *Tenodera aridifolia* | AF423805 | *Tenodera aridifolia* | AB598714 | AB596913 | AB597604 | *Tamolanica tamolana* | NC_007702 |
| Blattaria | Ectobiidae | *Ectobius lapponicus* | EU426877 | *Blattella germanica* | AF005243 | *Blattella nipponica* | AB598715 | AB596914 | AB597605 | *Blattella germanica* | NC_012901 |
| Isoptera | Termopsidae | *Zootermopsis angusticollis* | AY859614 | *Hodotermopsis japonica* | AY521856 | *Reticulitermes speratus* | AB598716 | AB596915 | AB597606 | *Zootermopsis angusticollis* | NC_018123 |
| Thysanoptera | Terebrantia | *Frankliniella intonsa* | JQ259051 | *Frankliniella intonsa* | JQ259051 | *Thrips palmi* | AB598717 | AB596916 | AB597607 | *Frankliniella intonsa* | JQ917403 |
| | Tubulifera | *Haplothrips chinensis* | JQ259052 | *Haplothrips chinensis* | JQ259052 | *Gynaikothrips ficorum* | AB598718 | AB596917 | AB597608 | *Haplothrips aculeatus* | NC_027488 |
| Hemiptera | Aphididae | *Aphis glycines* | JQ259057 | *Acyrthosiphon pisum* | U27819 | *Uroleucon nigrotuberculatum* | AB598722 | AB596921 | AB597612 | *Acyrthosiphon pisum* | NC_011594 |
| | Cicadidae | Cicadidae sp. NKU-013 | JQ309936 | *Okanagana utahensis* | U06478 | *Cryptotympana facialis* | AB598719 | AB596918 | AB597609 | *Gaeana maculata* | KM244671 |
| | Heteroptera | *Rhaphigaster nebulosa* | EU426880 | *Rhaphigaster nebulosa* | X89495 | *Anacanthocoris striicornis* | AB598720 | AB596919 | AB597610 | *Hydaropsis longirostris* | NC_012456 |
| Psocoptera | Psocidae | Cerastipsocinae sp. NKU-002 | JQ259058 | Cerastipsocinae sp. | JQ259054 | *Metylophorus* sp. | AB598723 | AB596922 | AB597613 | *Psococerastis albimaculata* | NC_021400 |
| Phthiraptera | Anoplura | *Haematopinus* sp. NKU-011 | JQ309932 | *Haematopinus* sp. | JQ309927 | *Pediculus humanus* | XM_002429812 | XM_002426633 | XM_002425468 | *Ibidoecus bisignatus* | NC_015999 |
| Hymenoptera | Apocrita | *Vespula pensylvanica* | AY859612 | *Vespula pensylvanica* | AY859613 | *Vespa analis insularis* | AB598725 | AB596924 | AB597615 | *Vespa mandarinia* | NC_027172 |
| | Symphyta | *Tenthredinidae gen.* sp. | EU414729 | *Notofenusa surosa* | GQ410584 | *Neocolochelyna itoi* | AB598724 | AB596923 | AB597614 | *Tenthredo tienmushana* | KR703581 |
| Megaloptera | Corydalidae | *Acanthacorydalis* sp. | JQ259049 | *Archichauliodes* sp. | EU815228 | *Protohermes grandis* | AB598726 | AB596925 | AB597616 | *Protohermes concolorus* | NC_011524 |
| Raphidioptera | Raphidiidae | *Raphidiidae sp. Sus1d* | GU169693 | *Mongoloraphidia martynovae* | EU815252 | *Inocellia japonica* | AB598727 | AB596926 | AB597617 | *Mongoloraphidia harmandi* | NC_013251 |
| Neuroptera | Mantispoidea | Mantispidae sp. NKU-006 | JQ259053 | *Zeugomantispa minuta* | EU815253 | *Chrysoperla nipponensis* | AB598728 | AB596927 | AB597618 | *Chrysoperla nipponensis* | NC_015093 |
| Strepsiptera | Mengenillidae | *Mengenilla sp. BMW-2012* | KC177631 | *Caenocholax fenyesi texensis* | DQ026302 | *Stichotrema asahinai* | AB598729 | AB596928 | AB597619 | *Mengenilla moldrzyki* | NC_018545 |
| Coleoptera | Polyphaga | *Tenebrio* sp. JMM-2003 | AY210843 | *Bolitophagus reticulatus* | EF362998 | *Tribolium castaneum* | XM_962197 | XM_968377 | XM_969560 | *Tribolium castaneum* | KM009121 |
| | Adephaga | Cicindelidae sp. | KJ825855* | *Cicindela aurulenta* | DQ337114 | *Eretes sticticus* | AB598730 | AB596929 | AB597620 | *Aspidytes niobe* | NC_012139 |
| Lepidoptera | Glossata | *Pieris napi* | EU414731 | *Anthocharis sara* | AF423785 | *Papilio polytes* | AB598732 | AB596931 | AB597622 | *Papilio polytes* | NC_024742 |
| Trichoptera | Integripalpia | Phryganeidae sp. NKU-007 | JQ259059 | *Pycnopsyche lepida* | AF286292 | *Stenopsyche marmorata* | AB598733 | AB596932 | AB597623 | *Eubasilissa regina* | NC_023374 |
| Diptera | Brachycera | *Drosophila melanogaster* | M21017 | *Drosophila melanogaster* | M21017 | *Drosophila melanogaster* | X88928 | NM_078569 | BT003265 | *Drosophila melanogaster* | NC_024511 |
| | Nematocera | *Anopheles albimanus* | L78065 | *Anopheles punctulatus* | AF121062 | *Anopheles gambiae* | XM_320778 | XM_317690 | XM_313416 | *Anopheles gambiae* | NC_002084 |
| Mecoptera | Boreidae | *Boreus hyemalis* | EU426882 | *Boreus* sp. NC-1995 | X89487 | *Panorpa takenouchii* | AB598734 | AB596933 | AB597624 | *Neopanorpa pulchra* | NC_013180 |
| Siphonaptera | Pulicidae | *Ctenocephalides felis* | FJ040538 | *Archaeopsylla erinacei* | X89486 | *Ctenocephalides felis* | AB598735 | AB596934 | AB597625 | *Jellisonia amadoi* | NC_022710 |

# Table S4. Fossil record and prior distributions for different analyses.

| Calibration Nodes | Fossils | Age (Ma) | BEAST analyses | | | Bayesian analysis | References |
|---|---|---|---|---|---|---|---|
| | | | ±one Stage/Age | Within certain Stage/Age | ±two Stage/Age | | |
| Root | *Rhyniognatha hirsti* | Pragian (410.8±2.8 - 407.6±2.6) | Normal Mean=465.15, SD=28.3 | Normal Mean=465.15, SD=28.3 | Normal Mean=465.15, SD=28.3 | -- | Engel and Grimaldi, 2004 |
| Amphiesmenoptera | *Archaeolepis mane* | Sinemurian (199.3±0.3 - 190.8±1.0) | Normal mean=194.7, SD=3.3 | Normal mean=194.7, SD=2.4 | Normal mean=194.7, SD=6.8 | Min=189.8, Max=411 | Whalley, 1985, 1986 |
| Blattodea | *Valditermes brenanae* | Hauterivian (132.9-129.4) | Normal Mean=131.15, SD=2.9 | Normal Mean=131.15, SD=0.8 | Normal Mean=131.15, SD=6.9 | Min=129.4, Max=411 | Jarzembowski, 1981; Engel et al., 2009 |
| Coleopterida = (Strepsiptera+Coleoptera) | *Triaplus sibiricus* | Changhsingian-Induan (254.14±0.07 - 251.2) | Normal Mean=252.71 SD=2.8 | Normal Mean=252.71, SD=0.7 | Normal Mean=252.71, SD=5.0 | Min=251.2, Max=411 | Volkov, 2013 |
| Dicondylia | *Rhyniognatha hirsti* | Pragian (410.8±2.8 - 407.6±2.6) | Normal Mean=409.3, SD=6.7 | Normal Mean=409.3, SD=2.6 | Normal Mean=409.3, SD=7.8 | Min=405, Max=521 | Engel and Grimaldi, 2004 |
| Diptera | *Archilimonia vogesiana* | Anisian (247.2 - 242.0) | Normal Mean=244.6, SD=3.5 | Normal Mean=244.6, SD=1.2 | Normal Mean=244.6, SD=3.7 | Min=242.0, Max=411 | Krzemiński and Krzemińska, 2003; Zhang, 2011 |
| Eukinolabia = (Embiodea+Phasmatodea) | *Cretophasmomima melanogramma* | Barremian-Early Aptian (129.7 ± 0.5 - 122.1 ± 0.3) | Normal Mean=126, SD=3.4 | Normal Mean=126 SD=2.0 | Normal Mean=126, SD=6.8 | Min=121.8, Max=411 | Wang et al., 2014 |
| Ephemeroptera | *Triassonurus doliiformis* | Anisian (247.2 - 242.0) | Normal Mean=244.6, SD=3.5 | Normal Mean=244.6, SD=1.2 | Normal Mean=244.6, SD=3.7 | Min=242.0, Max=521 | Sinitshenkova and Marchal-Papier, 2005 |
| Hemiptera | *Aviorrhyncha magnifica* | Moscovian (315.2±0.2- 307.0±0.1) | Normal Mean=311.15, SD=3.9 | Normal Mean=311.15, SD=2.1 | Normal Mean=311.15, SD=6.9 | Min=306.9, Max=411 | Nel et al., 2013 |
| Hymenoptera | *Triassoxyela foveolata* | Carnian (237.0 – 227.0) | Normal Mean=232.0, SD=5.3 | Normal Mean=232.0, SD=2.3 | Normal Mean=232.0, SD=7.5 | Min=227.0, Max=411 | Rasnitsyn, 1964; Ronquist et al., 2012 |
| Neuropterida | *Elmothone martynovae* | Artinskian – Kungurian (290.1±0.26 - 272.3±0.5) | Normal Mean=281.08, SD=6.6 | Normal Mean=281.08, SD=4.5 | Normal Mean=281.08, SD=8.8 | Min=271.8, Max=411 | Carpenter, 1976 |
| Odonata | *Triassothemis mendozensis* | Carnian (237.0 - 227.0) | Normal Mean=232, SD=5.3 | Normal Mean=232, SD=2.3 | Normal Mean=232, SD=7.5 | Min=227.0, Max=521 | Carpenter, 1960 |
| Orthoptera | *Raphogla rubra* | Artinskian- Kungurian (290.1±0.26 - 272.3±0.5) | Normal Mean=281.08, SD=6.6 | Normal Mean=281.08, SD=4.5 | Normal Mean=281.08, SD=8.8 | Min=271.8, Max=411 | Béthoux et al., 2002 |
| Psocodea | *Paramesopsocus adibi* | Callovian-Oxfordian (166.1±1.2 - 157.3±1.0) | Normal Mean=161.8, SD=4.0 | Normal Mean=161.8, SD=2.5 | Normal Mean=161.8, SD=4.8 | Min=156.3, Max=411 | Azar et al., 2008; Mockford et al., 2013 |
| Thysanoptera | *Tethysthrips libanicus* | Barremian (129.4 - 125.0) | Normal Mean=127.2, SD=2.8 | Normal Mean=127.2, SD=0.9 | Normal Mean=127.2, SD=6.5 | Min=125.0, Max=411 | Nel et al., 2010 |
| Notoptera | *Juramantophasma sinica* | Callovian – Oxfordian (166.1±1.2 - 157.3±1.0) | Normal Mean=161.8, SD=4.0 | Normal Mean=161.8, SD=2.5 | Normal Mean=161.8, SD=4.8 | Min=156.3, Max=411 | Huang et al., 2008 |

**File S5. Detailed phylogenetic justification and age justification for each fossil item.**

**i) Fossil specimen:** *Rhyniognatha hirsti* Tillyard, 1928

**Phylogenetic justification:**

The anterior mandibular articulation of *R. hirsti* forms an acetabulum (socket) on the inner angle of the appendage. This trait demonstrates that they are dicondylic, a mandibular form known only within the insects and corresponding to a monophyletic lineage comprising the Zygentoma and Pterygota. The mandibles of *Rhyniognatha* also are of the cutting form found only in metapterygotes (= Odonatoptera + Neoptera), a result of the close position of the posterior condyle to the anterior acetabulum and restructuring of the mandible into a more triangular form, demonstrating that *Rhyniognatha* possessed wings (Engel and Grimaldi 2004). Thus, characters of its mandibular form implies "metapterygote" affinities and accordingly crown-group membership within Insecta (and even within Pterygota).

**Age justification:**

*Rhyniognatha hirsti* is found in the Rhynie Chert of Aberdeenshire, Scotland. Spore assemblages that stratigraphically underly the Rhynie Chert are dated to the early Pragian to early Emsian (Wellman 2004, 2006; Parry et al., 2011; Wolfe et al., 2016). Based on this evidence a minimum age constraint may be defined at 405.0 Ma for the Rhynie Chert, using the Pragian-Emsian boundary (407.6 Ma ± 2.6 Myr) as a reference.

ii) **Fossil specimen:** *Triassonurus doliiformis* Sinitshenkova et al., 2005

**Phylogenetic justification:**

*Triassonurus doliiformis* has morphological features suggesting an affinity with the modern family Siphlonuridae, specifically large, non-flattened nymphs with a head longer than the prothorax, a massive mesothorax and considerably reduced metathorax, broad forewing pads that nearly completely cover the hind pads, abdominal segments lacking sharp denticles, large and rounded tergaliae, and elongate cerci and paracercus (Sinitshenkova et al., 2005; Wolfe et al., 2016). Siphlonuridae are within crown-group Ephemeroptera (Ogden et al., 2009).

**Age justification:**

*Triassonurus doliiformis* was found in the Grès-a-Voltzia Formation, Vosges, northeastern France (Gall and Grauvogel-Stamm, 1993). Sequence stratigraphy correlates the Grès-a-Voltzia Formation to the middle Anisian stage (Bourquin et al., 2006, 2007), the upper boundary of which is ca. 242 Ma (Cohen et al., 2013), and which may be used as a minimum age (Wolfe et al., 2016).

**iii) Fossil specimen:** *Triassothemis mendozensis* Carpenter, 1960.

**Phylogenetic justification:**

*Triassothemis mendozensis* is the earliest species of the extinct family Triassolestidae (Nicholson et al., 2015). Davis et al. (2011) support the placement of Triassolestidae within crown-group Epiprocta (= Anisozygoptera + Anisoptera) based on combination of molecular data and morphological characters.

**Age justification:**
*Triassothemis mendozensis* was recovered from the Potrerillos Formation of Mendoza, Argentina (Martins-Neto et al., 2008). U-Pb dating of zircons from the uppermost tuff layer of the formation provides an age of 230.3 Ma ± 2.3 Myr, or Carnian (Spalletti et al., 2009), and allowing for a minimum age of 228.0 Ma for *T. mendozensis* (Wolfe et al., 2016).

**iv) Fossil specimen:** *Raphogla rubra* Béthoux et al., 2002
**Phylogenetic justification:**
*Raphogla rubra* likely belongs to the stem of Gryllidea + Tettigoniidea, as evidenced by the broad area between Sc and the anterior wing margin; the moderately long Rs basal to the fusion with MA1a; and MP + CuA1 with merely one simple, anterior branch (Béthoux et al., 2002). Both Gryllidea and Tettigoniidea belong to the crown-group of Ensifera and accordingly *R. rubra* belongs to crown-group Orthoptera.
**Age justification:**
*Raphogla rubra* was uncovered from site F21D of the Salagou Formation in Hérault, France (Béthoux et al., 2002). U-Pb dating of zircons from tuff beds near the lower two-thirds of formation, corresponding to the Octon Member, recovered an age in the Artinskian (Michel et al., 2015). Based on this dating, the overlying Mérifrons Member has been inferred to start in the Artinskian and continuing into the Kungurian (Michel et al., 2015). Unfortunately, the precise layer of F21D within the Mérifrons Member is not known (Michel et al., 2015). Accordingly, we have followed Wolfe et al. (2016) and conservatively employed the upper boundary of the Kungurian (272.3 Ma ± 0.5 Myr) as a minimum age.

**v) Fossil specimen:** *Juramantophasma sinica* D. Huang et al., 2008
**Phylogenetic justification:**
*Juramantophasma sinica* shares traits with crown-group Mantophasmatodea, a suborder within Notoptera. Of particular importance is the presence of an elongate, dorsal process on the third tarsomere, the orthogonal orientation of the distal tarsomere relative to the preceding tarsomeres, the presence of enlarged and fan-shaped pretarsal arolia, and short, claw-like gonoplacs (Huang et al., 2008). The unsegmented cerci excludes the species from crown-group Grylloblattodea and a conservative placement for *J. sinica* is in the stem-group to Mantophasmatodea. Thus, the species at least belongs to Notoptera (=Grylloblattodea + Mantophasmatodea) (Wolfe et al., 2016).
**Age justification:**
*Juramantophasma sinica* is from the Jiulongshan Formation of the famous Daohugou Beds in Inner Mongolia, China (Huang et al., 2008). 40Ar/39Ar and U-Pb radiometric dating of ignimbrites has recovered dates from 165 Ma ± 2.5 Myr to 158.7 Ma ± 0.6 Myr (e.g., Chang et al., 2009a; Peng et al., 2012). Given that the fossiliferous shales overlay the volcanic deposits (Gao and Ren, 2006), they are correspondingly younger in age. The fossil beds correlate with Oxfordian sediments in Kazakhstan and China

(Zhang, 2015). Wolfe et al. (2016) have provided a conservative minimum age based on the radiometric evidence at 158.1 Ma (Oxfordian).

**vi) Fossil specimen:** *Cretophasmomima melanogramma* Wang et al., 2014
**Phylogenetic justification:**
*Cretophasmomima melanogramma* shares a strong twig-like appearance resulting from an elongation of the meso- and metathoracic segments with Phasmatodea (Wang et al., 2014). However, the lack of synapomorphies with crown-Phasmatodea such as the vomer and forceps-like extensions of the 10th abdominal tergum, indicate that it is likely part of stem-group Phasmatodea (Wang et al., 2014), and certainly within crown-Eukinolabia.
**Age justification:**
This fossil was discovered in the Yixian Formation, Liutiaogou Village, Ningcheng Country, Inner Mongolia, China (Wang et al., 2014). The Yixian Formation sits beneath the Jiufotang Formation and overlays the Tuchengzi Formation (Chang et al., 2009b; Zhou, 2006). 40Ar/39Ar radiometric dating has given ages of 129.7 Ma ± 0.5 Myr for basalts from Yixian Formation's bottom, and 122.1 Ma ± 0.3 Myr for tuff layers at the bottom of the Jiufotang Formation (Chang et al., 2009b: see also Zhou, 2006). A conservative minimum age of 121.8 Ma is appropriate for fossils from the Yixian Formation (Wolfe et al., 2016).

**vii) Fossil specimen:** *Valditermes brenanae* Jarzembowski, 1981
**Phylogenetic justification:**
*Valditermes brenanae* has been included in cladistic studies of morphological data and a placement in the stem of Mastotermitidae was recovered (Engel et al., 2009, 2016). Given that Mastotermitidae is the surviving sister group to all other Isoptera (Krishna et al., 2013; Djernæs et al., 2015), the species may be considered as part of the crown-group to Dictyoptera, and more precisely as crown-group Isoptera.
**Age justification:**
The holotype of *V. brenanae* originated from the Clockhouse Brickworks, Surrey, England (Jarzembowski, 1981), and from fossiliferous concretions of the Weald Clay Formation (Anderson, 1985). Wolfe et al. (2016) have summarized that among included ostracods the species *Cytheridea tuberculata* indicates placement in the Lower Weald Clay, at BGS Bed 3a (Anderson, 1985; Ross and Cook, 1995), and that based on palynomorphs the boundary between the Lower and Upper Weald Clay corresponds to the Hauterivian-Barremian boundary (Ross and Cook, 1995). The upper boundary of the Hauterivian (~129.4 Ma: Cohen et al., 2013), may therefore serve as a minimum age.

**viii) Fossil specimen:** *Tethysthrips libanicus* Nel et al. 2010
**Phylogenetic justification:**
*Tethysthrips libanicus* can be placed among the 'higher' Thripidae owing to the presence of forked and long 'trichomes' on the third and fourth antennomeres (Mound et al. 1980; Nel et al., 2010). *Tethysthrips* also share a narrowed forewing, the absence

of a pretarsal hamus, well-defined abdominal pleurites, and a well-developed, down-curved ovipositor (Nel et al., 2010).

**Age justification:**

*Tethysthrips libanicus* is represented by an amber inclusion from the Mdeyrij-Hammana outcrop, Baabda District, Lebanon (Nel et al., 2010). Maksoud et al. (in press) demonstrate an early Barremian age for the amber-bearing sediments, and, like Wolfe et al. (2016), we use a minimum age of 125.0 Ma.

**ix) Fossil specimen:** *Aviorrhyncha magnifica* Nel et al., 2013

**Phylogenetic justification:**

*Aviorrhyncha magnifica* has two synapomorphies of Euhemiptera (i.e., the group sister to Sternorrhyncha and containing all remaining extant Hemiptera): presence of an ambient vein and a well-developed concave CP (Nel et al., 2013). In contrast to most extant Euhemiptera, cua-cup is not zigzagged, and accordingly Nel et al. (2013) considered *A. magnifica* as stem-group Euhemiptera.

**Age justification:**

The species was discovered at the Terril No. 7 locality of the Avion outcrop, in Pas-de-Calais, France (Nel et al., 2013). This outcrop has been dated to Westphalian C/D (Bolsovian/Asturian), equivalent to the uppermost Moscovian of the Pennsylvanian (Nel et al., 2013; Richards, 2013). This allows for a minimum age of 306.9 Ma, using the upper boundary of the Moscovian (307.0 ± 0.1 Ma: Cohen et al., 2013).

**x) Fossil specimen:** *Paramesopsocus adibi* Azar et al. 2008

**Phylogenetic justification:**

*Paramesopsocus adibi* has the typically sclerotized and thickened pterostigma of the family Paramesopsocidae (Azar et al., 2008). The presence in the wing of well-developed veins Sc and A2 demonstrate that *Paramesopsocus* do not belong to Psocomorpha (Azar et al., 2008), and Mockford et al. (2013) have assigned the species to Electrentomidae.

**Age justification:**

This fossil was found at Karatau in southern Kazakhstan, and in the lacustrine-siltstones of the Karabastau Formation (Callovian/Oxfordian). The upper boundary of the Oxfordian is 157.3±1.0 Ma (Cohen et al., 2013), and provides a minimum age at 156.3 Ma.

**xi) Fossil specimen:** *Triassoxyela foveolata* Rasnitsyn, 1964

**Phylogenetic justification:**

*Triassoxyela foveolata* was recovered as stem-group Xyelidae in the cladistic analysis of Ronquist et al. (2012), but overall the wing venation is plesiomorphic for Xyelomorpha (Rasnitsyn, 1964, 1980), particularly in the retention of all primary veins. Nonetheless, the venation is overall characteristic for Hymenopterida (Rasnitsyn, 1964).

**Age justification:**

As elaborated by Wolfe et al. (2016), *T. foveolata* was found in the Madygen Formation, near the Fergana Valley, Kyrgyzstan. Paleobotanical evidence correlates the Madygen to the upper Keuper lithographic unit (Ladinian-Carnian: Dobruskina, 1995), and with the Cortaderitian Stage of Gondwana owing to the abundance of *Scytophyllum* (Morel et al., 2003). Wolfe et al. (2016) summarize the radiometric evidence that supports a minimum age of 227 Ma (Carnian upper boundary).

**xii) Fossil specimen:** *Elmothone martynovae* Carpenter, 1976
**Phylogenetic justification:**
Elmothone martynovae belongs to the extinct family Permithonidae. Morphological characters demonstrate placement of Permithonidae (=Protoneuroptera of Grimaldi and Engel, 2005), as the sister group to Neuroptera (Ren et al., 2009). Although permithonid monophyly is inconclusive and placement of other permithonids is obscure (Prokop et al., 2015), the presence of a straight apex to vein RA vein at the minimum establishes the species as part of this group (see also Wolfe et al., 2016).
**Age justification:**
This fossil was found at Elmo, Dickinson County, Kansas and in the Carlton Limestone Member of the Wellington Formation (Zambito et al., 2012; Prokop et al., 2015). Wolfe et al. (2016) note that the insect-bearing layer is correlated with the Leonardian regional Stage (Zambito et al., 2012), and on the basis of conchostracan biostratigraphy (Tasch, 1962). Given that the Leonardian spans the Artinskian and Kungurian Stages (Henderson et al., 2012), and that the upper boundary of the latter is at 272.3 Ma ± 0.5 Myr, a conservative minimum age of 271.8 Ma is employed (Wolfe et al., 2016).

**xiii) Fossil specimen:** *Triaplus sibiricus* Volkov, 2013
**Phylogenetic justification:**
*Triaplus sibiricus* belongs to the extinct family Triaplidae owing to the following traits: elongate beetle with comparatively smooth elytra, lacking punctate grooves; procoxae contiguous, without intercoxal process; metacoxae completely separating metaventrite from abdomen, long, with large metafemoral plates covering basal abdominal sterna; length of metacoxa subequal to its width (Volkov, 2013). Putative apomorphies of Adephaga, but excluding Triaplidae and Gyrinidae, are the presence of a ventral procoxal joint and a posteriorly elongate prosternal process (Beutel et al., 2013), and their absence are presumably plesiomorphies for Triaplidae.
**Age justification:**
The fossil was discovered in the Mal’tseva Formation, Babii Kamen, Novokuznetsk District, Kemerovo Region, Russia (Volkov, 2013). The Babii Kamen locality sits slightly above the Permian-Triassic boundary and the boundary of the Induan, at ~251.2 Ma (Cohen et al., 2013), is used as a minimum age.

**xiv) Fossil specimen:** *Archaeolepis mane* Whalley, 1985
**Phylogenetic justification:**

The holotype specimen of *A. mane* is an isolated hind wing, preserving some venation but more importantly bearing scales, the latter of which excludes the species from crown-group Trichoptera (Whalley, 1985, 1986). The scales demonstrate placement in Amphiesmenoptera and the presence of a single Sc branch in the venation of *A. mane* excludes the species from the groundplan of Lepidoptera (Kukalová-Peck and Willmann, 1990; Minet et al., 2010; Sukatsheva and Vassilenko, 2011). Placement as crown-Lepidoptera cannot be determined (Schachat and Brown, 2015), but placement among the stem-group is likely based on the hind wing scales (Whalley, 1986).

**Age justification:**

*Archaeolepis mane* was found in the 'Shales with Beef' (Lang et al., 1923), and specifically a calcareous flatstone layer of the *Caenisites turneri* ammonoid Zone (Sohn et al., 2012). Chemostratigraphic evidence places this zone within the middle Sinemurian (Jenkyns et al., 2002), and the upper boundary provides a minimum age of 195.3 Ma (Wolfe et al., 2016).

**xv) Fossil specimen:** *Archilimonia vogesiana* Krzemiński and Krzemińska, 2003

**Phylogenetic justification:**

Although originally placed in an extinct family as Archilominiidae (Krzemiński and Krzemińska, 2003), the species has been supported within Pediciidae, a family placed by Petersen et al. (2010) as sister to all other Tipuloidea. Pediciidae have many morphological features considered similar to the tipuloid groundplan: a large, well-sclerotized larval head capsule, presence of ommatrichia, and a Sc2 vein proximal to Rs in the wing (Oosterbroek & Theowald, 1991; Starý, 1992; Petersen et al., 2010).

**Age justification:**

The fossil was found at Bust, Vosges, France, and in an Aegean delta plain claystone/siltstone of the Gres-a-Voltzia Formation. Bourquin et al. (2006, 2007) correlate the Grès-a-Voltzia Formation to the middle Anisian based on sequence stratigraphy. We therefore use the upper boundary of the Anisian at ~242 Ma (Cohen et al., 2013) as a minimum age.

**xvi) Fossil specimen:** *Yicaris dianensis* Zhang et al., 2007

**Phylogenetic justification:**

Phylogenetic morphological analyses as well as simultaneous analyses with DNA sequence data place *Y. dianensis* within crown-group Pancrustacea (Edgecombe, 2010; Legg et al., 2013; Oakley et al., 2013; Wolfe and Hegna, 2014). Of particular importance are the presence of epipodites on the thoracic appendages; paddle-shaped mandibular and maxillary exopods; and the protopodite of post-mandibular appendages formed as soft, setiferous endites (Wolfe et al., 2016).

**Age justification:**

*Yicaris dianensis* was recovered from the Yu'anshan Formation in Yunnan Province, China and has been attributed to the *Eoredlichia-Wutingaspis* Biozone (Zhang et al., 2007), although this biozone is no longer recognized (Peng, 2003, 2009). *Eoredlichia* are known to co-occur with *Hupeidiscus*, and this is diagnostic of the

*Hupeidiscus-Sinodiscus* Biozone — a formally recognized biozone of the Nangaoan Stage in China (Peng and Babcock, 2008). The Nangaoan Stage is the third stage of the Cambrian System (Peng et al., 2012), therefore a maximum constraint can be placed for its lower boundary at 521 Ma (Peng et al., 2012; Peng and Babcock, 2008; Wolfe et al., 2016).

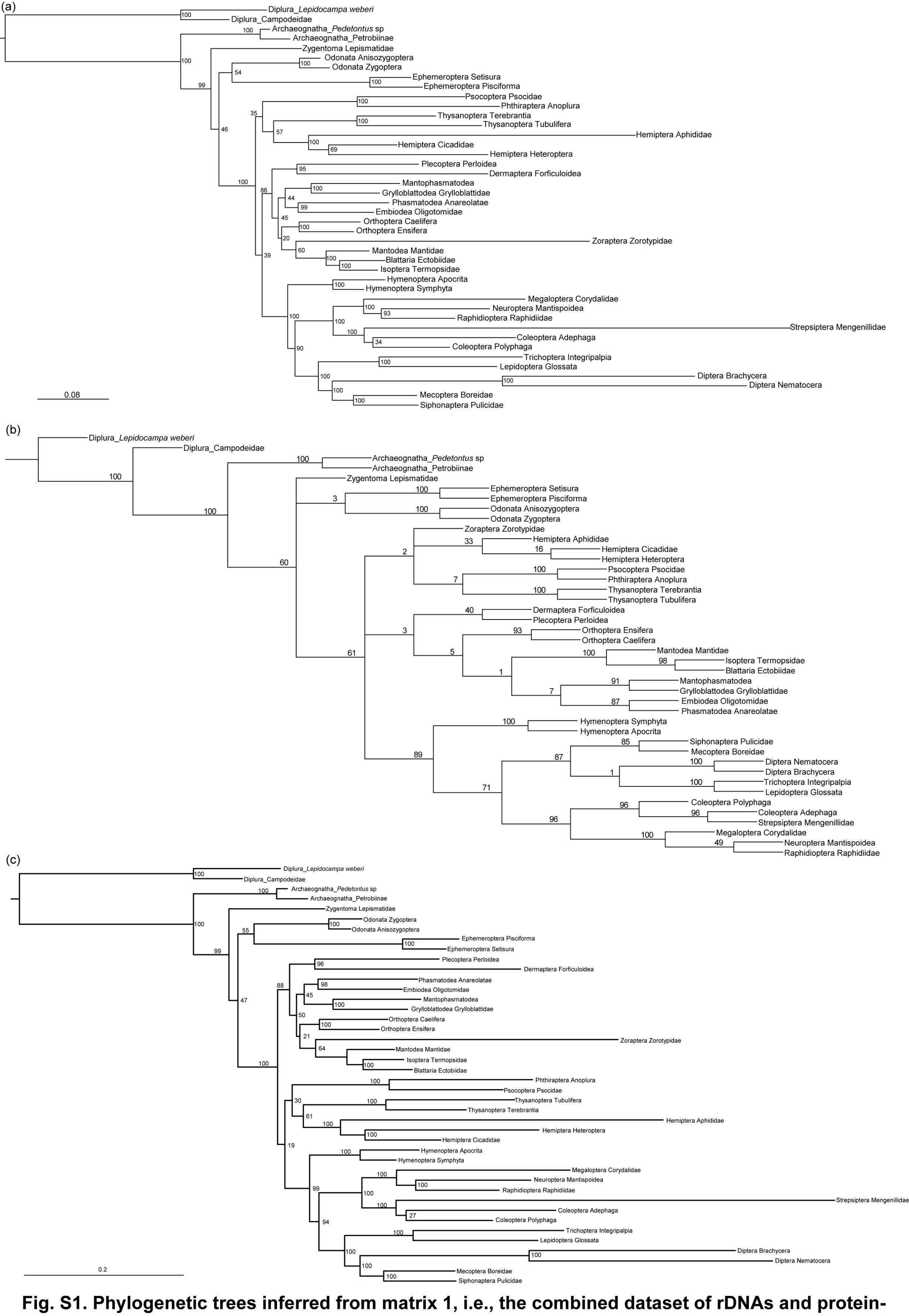


**Fig. S1. Phylogenetic trees inferred from matrix 1, i.e., the combined dataset of rDNAs and protein-coding genes.** A) ML topology with DNA model applied to the paired sites of rDNAs, B) MP topology, C) ML topology with Doublet model applied to the paired sites of rRNAs. Posterior probabilities and bootstrap values are shown at each node, respectively.

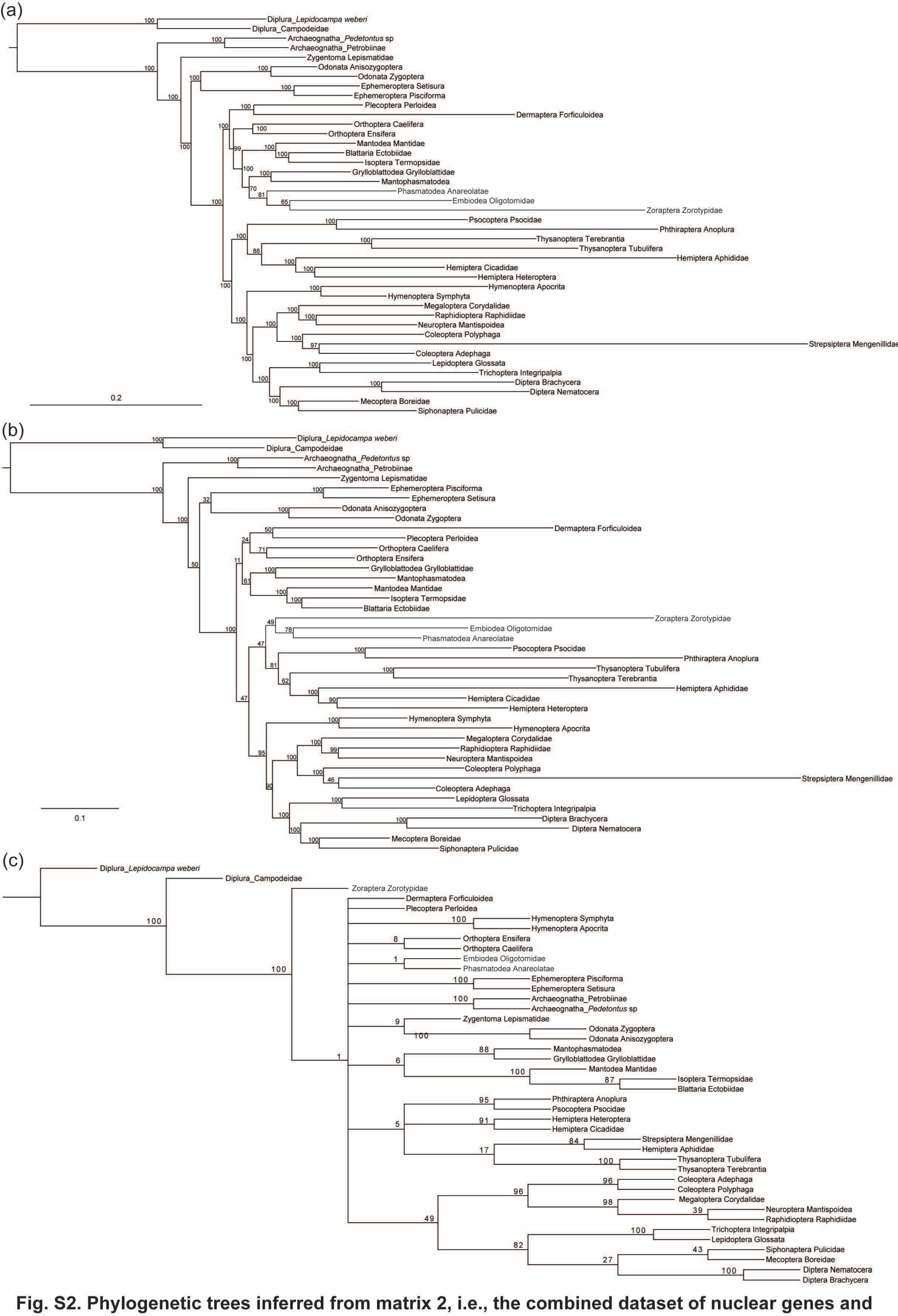


**Fig. S2. Phylogenetic trees inferred from matrix 2, i.e., the combined dataset of nuclear genes and the first codon positions of mitochondrial PCGs.** A) BI topology, B) ML topology, C) MP topology. Posterior probabilities and bootstrap values are shown at each node, respectively.

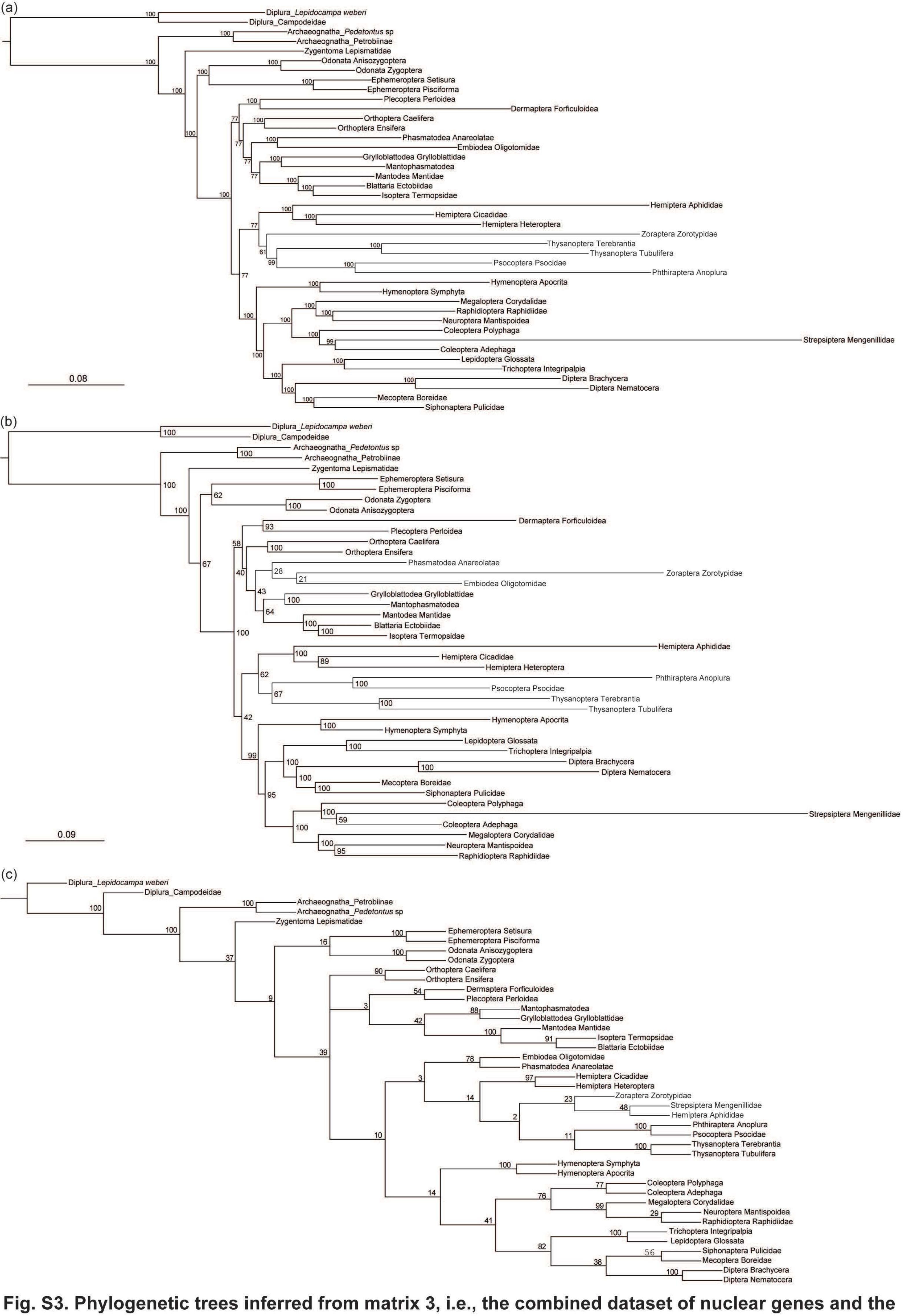


**Fig. S3. Phylogenetic trees inferred from matrix 3, i.e., the combined dataset of nuclear genes and the second codon positions of Mitochondrial PCGs.** A) BI topology, B) ML topology, C) MP topology. Posterior probabilities and bootstrap values are shown at each node, respectively.

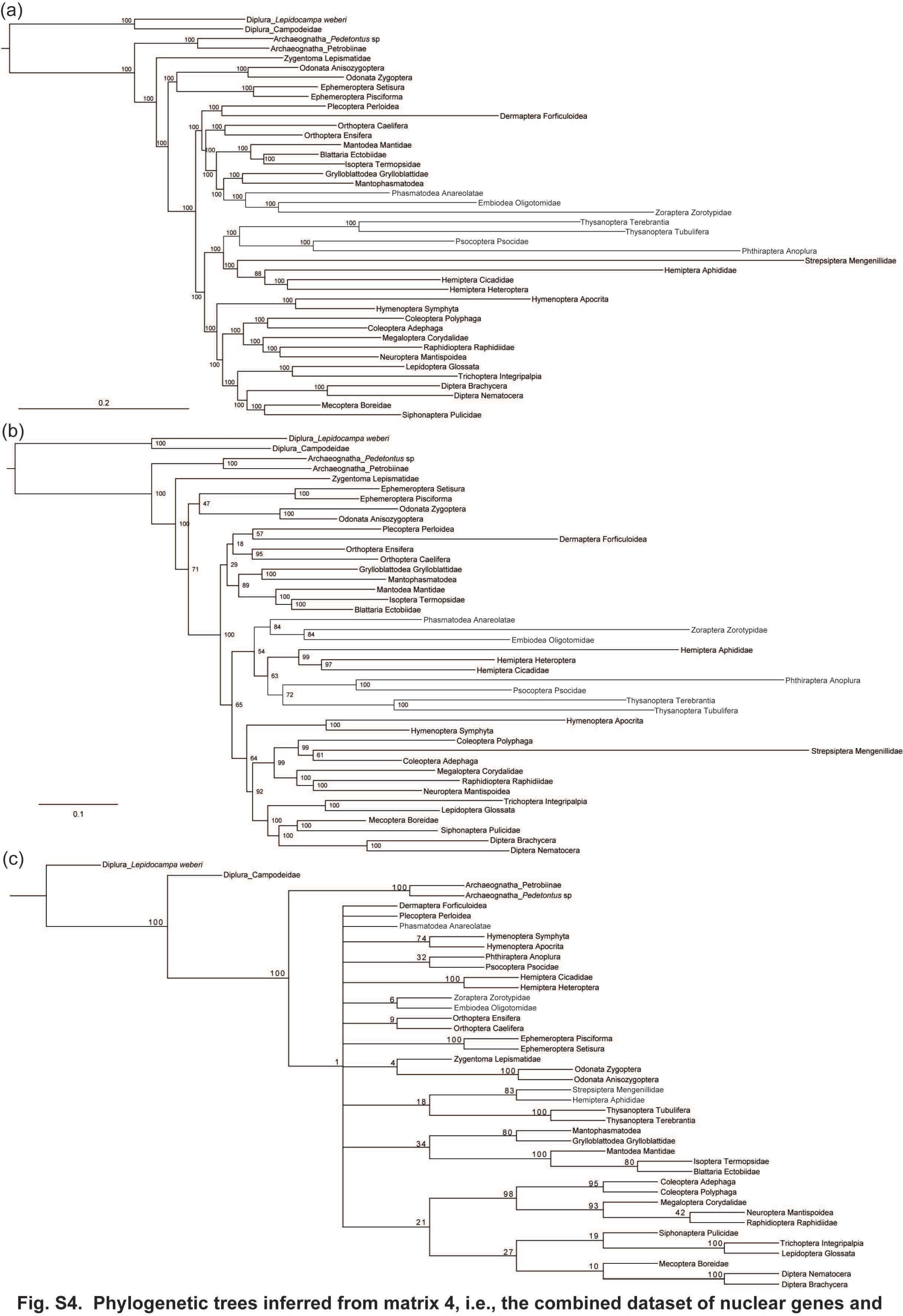


**Fig. S4. Phylogenetic trees inferred from matrix 4, i.e., the combined dataset of nuclear genes and the first two codon positions of mitochondrial PCGs.** A) BI topology, B) ML topology, C) MP topology. Posterior probabilities and bootstrap values are shown at each node, respectively.

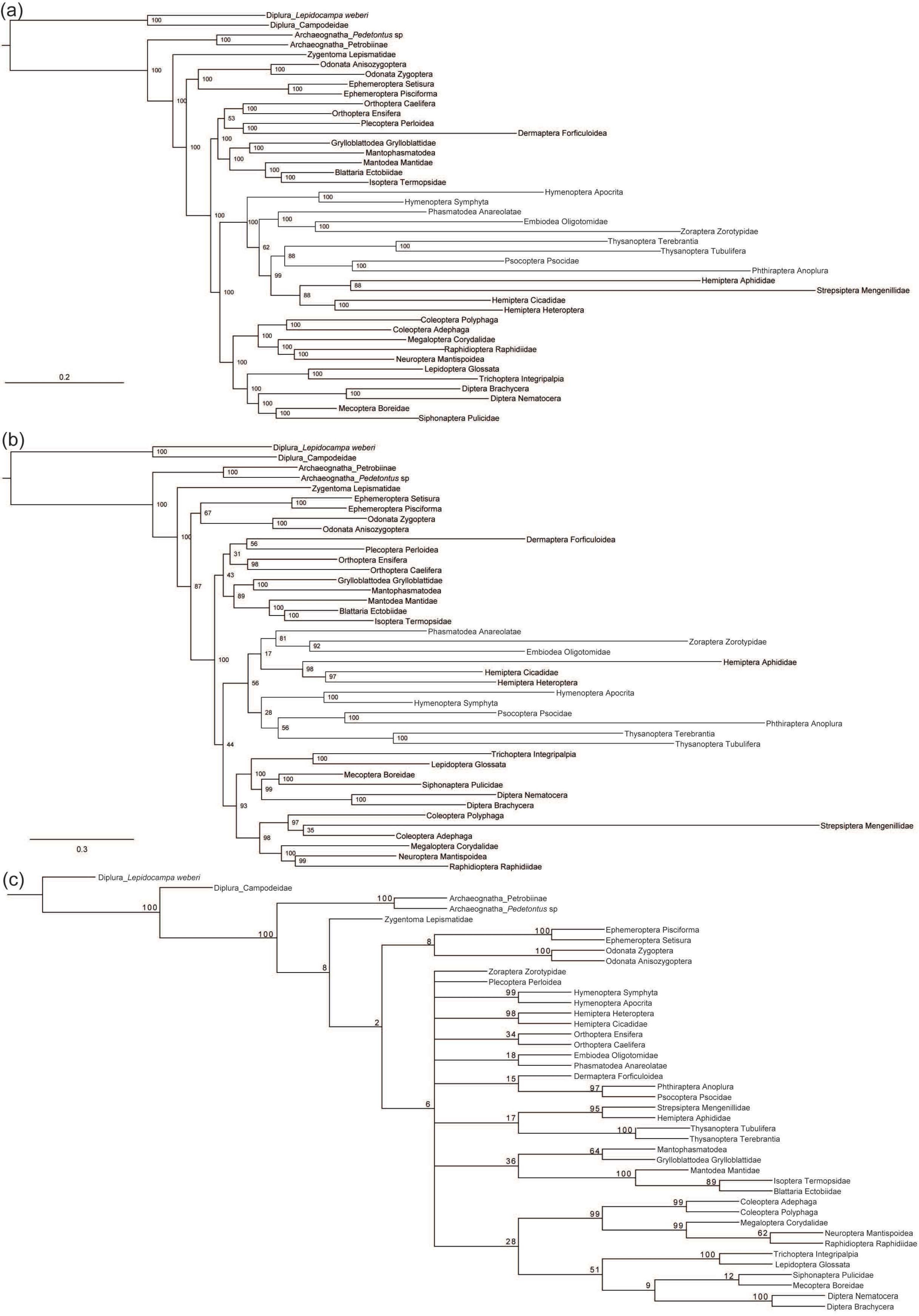


**Fig. S5. Phylogenetic trees inferred from matrix 5, i.e., the combined dataset of nuclear genes and the amino acid sequences of mitochondrial PCGs.** A) BI topology, B) ML topology, C) MP topology. Posterior probabilities and bootstrap values are shown at each node, respectively.

# References


Brauckmann, C. & Schneider, J. Ein unter-karbonisches Insekt aus dem Raum Bitterfeld/Delitzsch (Pterygota, Arnsbergium, Deutschland) [A Lower Carboniferous insect from the Bitterfeld/Delitzsch area (Pterygota, Arnsbergian, Germany)]. *Neues. Jahrb. Geol. P-M* 17–30 (1996).

Béthoux, O. & Nel, A. Venation pattern and revision of Orthoptera sensu nov. and sister groups. Phylogeny of Palaeozoic and Mesozoic Orthoptera sensu nov. *Zootaxa* **96,** 1–88 (2002)

Zhang, Z. J., Schneider, J. W. & Hong, Y. C. The most ancient roach (Blattodea): a new genus and species from the earliest Late Carboniferous (Namurian) of China, with a discussion of the phylomorphogeny of early blattids. *J. Syst. Palaeontol.* **11,** 27–40 (2013).

Peng, D., Hong, Y. & Zhang Z. Namurian insects (Diaphanopterodea) from Qilianshan Mountains, China. *Geological Bulletin of China* **24,** 219–234 (2005).

Nel, P., Azar, D., Prokop, J., Roques, P., Hodebert, G. & Nel, A. From Carboniferous to Recent: wing venation enlightens evolution of thysanopteran lineage. *J. Syst. Palaeontol.* **10,** 385–399 (2012).

Nel, A. *et al*. The earliest known holometabolous insects. *Nature* **503,** 257–261 (2013).

Nel, A., Roques, P., Nel, P., Prokop, J. & Steyer, J. S. The earliest holometabolous insect from the Carboniferous: a "crucial" innovation with delayed success (Insecta Protomeropina Protomeropidae). *Annales de la Société Entomologique de France* **43,** 349–355 (2007).

Engel, M. S. & Grimaldi, D. A. New light shed on the oldest insect. *Nature* **427,** 627–630 (2004).

Whalley, P. The systematics and palaeogeography of the Lower Jurassic insects of Dorset, England. *Bulletin of the British Museum of Natural History (Geology)* **39,** 107–189 (1985).

Whalley, P. A review of the current fossil evidence of Lepidoptera in the Mesozoic. *Biol. J. Linn. Soc.* **28**, 253–271 (1986).

Jarzembowski, E. A. An early Cretaceous termite from southern England (Isoptera: Hodotermitidae). *Syst. Entomol.* **6,** 91–96 (1981).

Engel, M. S., Grimaldi, D. A., Krishna, K. Termites (Isoptera): their phylogeny, classification, and rise to ecological dominance. *Am. Mus. Novit.* 1–27 (2009).

Volkov, A. N. New species of Triaplidae from the Babii Kamen' locality (Kuznetsk Basin). *Paleontol. J.* **47,** 94–97 (2013).

Krzemiński, W. & Krzemińska, E. Triassic Diptera: descriptions, revisions and phylogenetic relations. *Acta. Zool. Cracov.* **46,** 153–184 (2003).

Zhang, Z. Q. Animal biodiversity: An outline of higher-level classification and survey of taxonomic richness (Addenda 2013). *Zootaxa* **3703**, 1–82 (2013).

Wang, M., Béthoux, O., Bradler, S., Jacques, F.M.B., Cui, Y. & Ren, D. Under Cover at Pre-Angiosperm Times: A Cloaked Phasmatodean Insect from the Early Cretaceous Jehol Biota. *PLoS ONE* **9,** e91290 (2014).

Sinitshenkova, N. D. & Marchal-Papier, F. Systematic Paleontology, in The Ephemeridea (Insecta) from the Gres a Voltzia (early Middle Triassic) of the Vosges (NE France). *Palaeontol Z.* **79,** 378–397 (2005).

Nel, A. et al. The earliest known holometabolous insects. *Nature* **503,** 257–261 (2013).

Rasnitsyn, A. P. Novye Triasovye pereponchatokrylye sredney Azii. *Paleontologicheskii Zhurnal* **1964,** 88–96 (1964).

Ronquist, F., Klopfstein, S., Vilhelmsen, L., Schulmeister, S., Murray, D. L., Rasnitsyn, A. P. A Total-Evidence Approach to Dating with Fossils, Applied to the Early Radiation of the Hymenoptera. *Syst. Biol.* **61,** 973–999 (2012).

Carpenter, F. M. The Lower Permian Insects of Kansas: Part 12. Protorthoptera (continued), Neuroptera, Additional Palaeodictyoptera & Families of Uncertain Position. *Psyche* **83**, 336-376 (1976).

Carpenter, F. M. A Triassic Odonate from Argentina. *Psyche* **67,** 71–75 (1960).

Béthoux, O., Nel, A., Lapeyrie, J., Gand, G. & Galtier, J. *Raphogla rubra* gen. n. sp. n. the oldest representative of the clade of modern Ensifera (Orthoptera: Tettigoniidea, Gryllidea). *Eur. J. Entomol.* **99,** 111–116 (2002).

Azar, D., Hajar, L., Indary, C. & Nel, A. Paramesopsocidae, a new Mesozoic psocid

family (Insecta: Psocodea "Psocoptera": Psocomorpha). *Ann. Soc. Entomol. Fr. (N.S.)* **44,** 459–470 (2008).

Mockford, E. L., Lienhard, C. & Yoshizawa K. Revised classification of ‘Psocoptera’ from Cretaceous amber, a reassessment of published information. *Insecta Matsumarana* **69,** 1–26 (2013).

Nel, P., Peñalver, E., Azar, D., Hodebert, G. & Nel, A. Modern thrips families Thripidae and Phlaeothripidae in Early Cretaceous amber (Insecta: Thysanoptera). *Annales de la Société Entomologique de France* **46,** 154–163 (2010).

Huang, D. Y., Nel, A., Zompro, O. & Waller, A. Mantophasmatodea now in the Jurassic. *Naturwissenschaften* **95,** 947–952 (2008).

Anderson, F.W. 1985. Ostracod faunas in the Purbeck and Wealden of England. J. Micropalaeontology 4, 1–67.

Azar, D., Hajar, L., Indary C., Nel, A. 2008. Paramesopsocidae, a new Mesozoic psocid family (Insecta: Psocodea "Psocoptera": Psocomorpha). Annales de la Société Entomologique de France 44, 459–470.

Beutel, R.G., Wang, B., Tan, J-J, Ge, S-Q, Ren, D., Yang, X-K. 2013. On the phylogeny and evolution of Mesozoic and extant lineages of Adephaga (Coleoptera, Insecta). Cladistics 29, 147–165.

Béthoux, O., Nel, A. 2002. Venation pattern and revision of Orthoptera sensu nov. and sister groups. Phylogeny of Palaeozoic and Mesozoic Orthoptera sensu nov. Zootaxa 96, 1–88.

Bourquin, S., Durand, M., Diez, J. B., Broutin, J., Fluteau, F. 2007. El limite Permico-Triasico y la sedimentacion durante el Triasico inferior en las cuencas de Europa occidental: una vision general. J. Iber. Geol. 221–237.

Bourquin, S., Peron, S., Durand, M. 2006. Lower Triassic sequence stratigraphy of the western part of the Germanic Basin (west of Black Forest): Fluvial system evolution through time and space. Sediment. Geol. 186, 187–211.

Chang, S., Zhang, H., Renne, P. R., Fang, Y. 2009a. High-precision 40Ar/39Ar age constraints on the basal Lanqi Formation and its implications for the origin of angiosperm plants. Earth Planet. Sci. Lett. 279, 212–221.

Chang, S., Zhang, H., Renne, P.R., Fang, Y. 2009b. High-precision 40Ar/39Ar age for the Jehol Biota. Palaeogeogr. Palaeoclimatol. Palaeoecol. 280, 94–104.

Cohen, K. M., Finney, S. C., Gibbard, P. L., Fan, J. X. 2013. The ICS International Chronostratigraphic Chart. Episodes 36: 199–204.

Davis, R. B., Nicholson, D. B., Saunders, E. L., Mayhew, P. J. 2011. Fossil gaps inferred from phylogenies alter the apparent nature of diversification in dragonflies and their relatives. BMC Evol. Biol. 11, 252.

Djernæs, M., Klass, K. D., Eggleton, P. 2015. Identifying possible sister groups of Cryptocercidae + Isoptera: A combined molecular and morphological phylogeny of Dictyoptera. Mol. Phylogenet. Evol. 84, 284–303.

Dobruskina, I. A. 1995. Keuper (Triassic) Flora from Middle Asia (Madygen, Southern Fergana): Bulletin 5. New Mexico Museum of Natural History and Science, Albuquerque.

Edgecombe, G. D. 2010. Palaeomorphology: fossils and the inference of cladistic relationships. Acta Zool. 91, 72–80.

Engel, M.S., Barden, P., Riccio, M.L., & Grimaldi, D.A. 2016. Morphologically specialized termite castes and advanced sociality in the Early Cretaceous. Current Biology 26: 522–530.

Engel, M. S., Grimaldi, D. A. 2004. New light shed on the oldest insect. Nature 427: 627–630.

Engel, M. S., Grimaldi, D. A., Krishna, K. 2009. Termites (Isoptera): their phylogeny, classification, and rise to ecological dominance. Am. Mus. Novit. 1–27.

Gall, J.C., Grauvogel-Stamm, L. 1993. Buntsandstein (lower Triassic) of Eastern France. Nonmarine Triassic Bull. 3 3, 141.

Gao, K., Ren, D. 2006. Radiometric Dating of Ignimbrite from Inner Mongolia Provides no Indication of a Post-Middle Jurassic Age for the Daohugou Beds. Acta Geol. Sin. 80, 42–45.

Grimaldi, D., Engel, M.S., 2005. Evolution of the Insects. Cambridge University Press, Cambridge.

Henderson, C. M., Davydov and, V. I., Wardlaw, B. R., Gradstein, F. M., Hammer, O.

2012. The Permian Period, in: The Geologic Time Scale. Elsevier, pp. 653–679.
Huang, D., Nel, A., Zompro, O., Waller, A. 2008. Mantophasmatodea now in the Jurassic. Naturwissenschaften 95, 947–952.
Jarzembowski, E. A.  1981. An early Cretaceous termite from southern England (Isoptera: Hodotermitidae). Syst. Entomol. 6, 91–96
Jenkyns, H. C., Jones, C. E., Gröcke, D. R., Hesselbo, S. P., Parkinson, D. N. 2002. Chemostratigraphy of the Jurassic System: applications, limitations and implications for palaeoceanography. J. Geol. Soc. 159, 351–378.
Krishna, K., Grimaldi, D.A., Krishna, V., & Engel, M.S. 2013. Treatise on the Isoptera of the world. Bulletin of the American Museum of Natural History 377: 1–2704.
Krzemiński, W., Krzemińska, E. 2003. Triassic Diptera: descriptions, revisions and phylogenetic relations. Acta Zoologica Cracoviensia 46(Suppl):153–184.
Kukalová-Peck, J., Willmann, R. 1990. Lower Permian “mecopteroid-like” insects from central Europe (Insecta, Endopterygota). Can. J. Earth Sci. 27, 459–468.
Lang, W. D., Spath, L. F., Richardson, W. A. 1923. Shales-with-“beef,”a Sequence in the Lower Lias of the Dorset Coast. Q. J. Geol. Soc. 79, 47–66.
Legg, D. A., Sutton, M. D., Edgecombe, G. D. 2013. Arthropod fossil data increase congruence of morphological and molecular phylogenies. Nat. Commun. 4.
Maksoud, S., Azar, D., Granier, B., & Gèze, R. In press. New data on the age of the Lower Cretaceous amber outcrops of Lebanon. Palaeoworld.
Martins-Neto, R.G., Gallego, O.F., Zavattieri, A.M., 2008. The Triassic insect fauna from Argentina: Coleoptera, Hemiptera and Orthoptera from the Potrerillos Formation, south of cerro Cacheuta, Cuyana basin. Alavesia 2, 47–58.
Michel, L. A., Tabor, N. J., Montañez, I. P., Schmitz, M. D., Davydov, V. I. 2015. Chronostratigraphy and Paleoclimatology of the Lodève Basin, France: Evidence for a pan-tropical aridification event across the Carboniferous–Permian boundary. Palaeogeogr. Palaeoclimatol. Palaeoecol. 430, 118–131.
Minet, J., Huang, D.-Y., Wu, H., Nel, A. 2010. Early Mecopterida and the systematic position of the Microptysmatidae (Insecta: Endopterygota). Ann. Société Entomol. Fr. 46, 262–270.

Mockford, E. L., Lienhard, C., Yoshizawa, K. 2013. Revised classification of "Psocoptera" from Cretaceous amber, a reassessment of published information. Insecta Matsumurana 69:1–26.

Morel, E. M., Artabe, A. E., Spalletti, L. A. 2003. Triassic floras of Argentina: biostratigraphy, floristic events and comparison with other areas of Gondwana and Laurasia. Alcheringa 27, 231–243.

Mound L. A., Heming B. S., Palmer J. M. 1980. Phylogenetic relationships between the families of recent Thysanoptera. Zoological Journal of the Linnean Society 69: 111–141.

Nel, A., Roques, P., Nel, P., Prokin, A.A., Bourgoin, T., Prokop, J., Szwedo, J., Azar, D., Desutter-Grandcolas, L., Wappler, T., Garrouste, R., Coty, D., Huang, D., Engel, M.S., Kirejtshuk, A.G. 2013. The earliest known holometabolous insects. Nature 503, 257–261.

Nel, P., Peñalver, E., Azar, D., Hodebert, G., Nel, A. 2010. Modern thrips families Thripidae and Phlaeothripidae in Early Cretaceous amber (Insecta: Thysanoptera). Annales de la Société Entomologique de France 46:154–163.

Nicholson, D.B., Mayhew, P.J., Ross, A.J., 2015. Changes to the Fossil Record of Insects through Fifteen Years of Discovery. PLoS ONE 10, e0128554.

Oakley, T. H., Wolfe, J. M., Lindgren, A. R., Zaharoff, A. K. 2013. Phylotranscriptomics to Bring the Understudied into the Fold: Monophyletic Ostracoda, Fossil Placement, and Pancrustacean Phylogeny. Mol. Biol. Evol. 30, 215–233.

Ogden, T. H., Gattolliat, J. L., Sartori, M., Staniczek, A. H., Soldán, T., Whiting, M. F. 2009. Towards a new paradigm in mayfly phylogeny (Ephemeroptera): combined analysis of morphological and molecular data. Syst. Entomol. 34, 616–634.

Oosterbroek, P., Theowald, B. 1991. Phylogeny of the Tipuloidea based on characters of larvae and pupae (Diptera, Nematocera) with an index to the literature except Tipulidae. Tijdshcrift voor Entomologie, 134, 211–267.

Parry, S. F., Noble, S. R., Crowley, Q. G., Wellman, C. H. 2011. A high-precision U-Pb age constraint on the Rhynie Chert Konservat-Lagerstatte: time scale and other

implications. J. Geol. Soc. 168, 863–872.

Peng, N., Liu, Y., Kuang, H., Jiang, X., Xu, H. 2012. Stratigraphy and Geochronology of Vertebrate Fossil-Bearing Jurassic Strata from Linglongta, Jianchang County, Western Liaoning, Northeastern China. Acta Geol. Sin. 86, 1326–1339.

Peng, S. 2009. The newly-developed Cambrian biostratigraphic succession and chronostratigraphic scheme for South China. Chin. Sci. Bull. 54, 4161–4170.

Peng, S. 2003. Chronostratigraphic subdivision of the Cambrian of China. Geol. Acta 1, 135–144.

Peng, S., Babcock, L.E. 2008. Cambrian Period, in: The Concise Geologic Time Scale. Cambridge University Press, Cambridge, pp. 37–46.

Peng, S., Babcock, L. E., Cooper, R. A. 2012. The Cambrian Period, in: The Geologic Time Scale. Elsevier, pp. 437–488.

Petersen, M. J., Bertone, M. A., Wiegmann, B. M., Courtney, G.W. 2010. Phylogenetic synthesis of morphological and molecular data reveals new insights into the higher-level classification of Tipuloidea (Diptera). Syst. Entomol. 35, 526–545.

Prokop, J., Rodrigues Fernandes, F., Lapeyrie, J., Nel, A. 2015. Discovery of the first lacewings (Neuroptera: Permithonidae) from the Guadalupian of the Lodève Basin (Southern France). Geobios 48, 263–270.

Rasnitsyn, A. P. 1964. New Triassic Hymenoptera of the Middle Asia. Paleontol. Zhurnal 1, 88–96.

Rasnitsyn, A. P. 1980. Origin and evolution of Hymenoptera. Trudy Paleontologicheskogo Instituta Academii Nauk SSSR 174, 1–192.

Ren, D., Labandeira, C. C., Santiago-Blay, J. A., Rasnitsyn, A. P., Shih, C., Bashkuev, A., Logan, M. A. V., Hotton, C. L., Dilcher, D. L. 2009. A Probable Pollination Mode Before Angiosperms: Eurasian, Long-Proboscid Scorpionflies. Science 326, 840–846.

Richards, B. C. 2013. Current status of the International Carboniferous time scale. Carbonif.-Permian Transit. NM Mus Nat Hist Sci Bull 60, 348–353.

Ronquist, F., Klopfstein, S., Vilhelmsen, L., Schulmeister, S., Murray, D. L., Rasnitsyn, A. P. 2012. A Total-Evidence Approach to Dating with Fossils, Applied to the

Early Radiation of the Hymenoptera. Syst. Biol. 61, 973–999.

Ross, A. J., Cook, E. 1995. The stratigraphy and palaeontology of the Upper Weald Clay (Barremian) at Smokejacks Brickworks, Ockley, Surrey, England. Cretac. Res. 16, 705–716.

Schachat, S. R., Brown, R. L. 2015. Color Pattern on the Forewing of Micropterix (Lepidoptera: Micropterigidae): Insights into the Evolution of Wing Pattern and Wing Venation in Moths. PLOS ONE 10, e0139972.

Sinitshenkova, N. D., Marchal-Papier, F., Grauvogel-Stamm, L., Gall, J. C. 2005. The Ephemeridea (Insecta) from the Grès à Voltzia (early Middle Triassic) of the Vosges (NE France). Paläontol. Z. 79, 377–397.

Sohn, J. C., Labandeira, C. C., Davis, D. R., Mitter, C. 2012. An annotated catalog of fossil and subfossil Lepidoptera (Insecta: Holometabola) of the world. Zootaxa 3286, 1–132.

Spalletti, L. A., Fanning, M., Rapela, C. W. 2009. Dating the Triassic continental rift in the southern Andes: the Potrerillos Formation, Cuyo basin, Argentina. Geol. Acta 6, 267–283.

Starý, J. 1992. Phylogeny and classification of Tipulomorpha, with special emphasis on the family Limoniidae. Acta Zoologica Cracoviensia 35, 11–36.

Sukatsheva, I. D., Vassilenko, D. V. 2011. Caddisflies from Chernovskie Kopi (Jurassic/Cretaceous of Transbaikalia). Zoosymposia 5, 434–438.

Tasch, P. 1962. Vertical extension of mid-continent Leonardian insect occurrences. Science 135, 378–379.

Volkov, A. N. 2013. New species of Triaplidae from the Babii Kamen' locality (Kuznetsk Basin). Paleontological Journal 47, 94–97

Wang, M., Béthoux, O., Bradler, S., Jacques, F.M.B., Cui, Y., Ren, D., 2014. Under Cover at Pre-Angiosperm Times: A Cloaked Phasmatodean Insect from the Early Cretaceous Jehol Biota. PLoS ONE 9, e91290.

Wellman, C. H. 2004. Palaeoecology and palaeophytogeography of the Rhynie chert plants: evidence from integrated analysis of in situ and dispersed spores. Proceedings of the Royal Society of London, Series B 271, 985–992.

Wellman, C. H. 2006. Spore assemblages from the Lower Devonian 'Lower Old Red Sandstone' deposits of the Rhynie outlier, Scotland. Transactions of the Royal Society of Edinburgh: Earth Sciences, 97, 167–211.

Whalley, P. E. 1985. The systematics and palaeogeography of the Lower Jurassic insects of Dorset, England. Bull. Br. Mus. Nat. Hist. Geol. 39, 107–189.

Whalley, P. 1986. A review of the current fossil evidence of Lepidoptera in the Mesozoic. Biol. J. Linn. Soc. 28, 253–271.

Wolfe, J. M., Daley, A. C., Legg, D. A., Edgecombe, G. D. 2016. Fossil calibrations for the arthropod tree of life. Earth-Science Reviews 160: 43–110.

Wolfe, J. M., Hegna, T. A. 2014. Testing the phylogenetic position of Cambrian pancrustacean larval fossils by coding ontogenetic stages. Cladistics 30, 366–390.

Zambito, J. J., Benison, K. C., Foster, T., Soreghan, G. S., Kane, M., Soreghan, M. 2012. Lithostratigraphy of the Permian Red Beds and Evaporites in the Rebecca K. Bounds Core, Greeley County, Kansas. Kans. Geol. Surv. Open-File Rep. 2012, 45.

Zhang, J. 2015. Archisargoid flies (Diptera, Brachycera, Archisargidae and Kovalevisargidae) from the Jurassic Daohugou biota of China, and the related biostratigraphical correlation and geological age. J. Syst. Palaeontol. 13, 857–881.

Zhang, X., Siveter, D.J., Waloszek, D., Maas, A. 2007. An epipodite-bearing crown-group crustacean from the Lower Cambrian. Nature 449, 595–598.

Zhou, Z. 2006. Evolutionary radiation of the Jehol Biota: chronological and ecological perspectives. Geol. J. 41, 377–393.
